# Epidemiology, clinical outcomes, and treatment patterns of cytomegalovirus infection after allogeneic hematopoietic stem cell transplantation in China: a scoping review and meta-analysis

**DOI:** 10.3389/fmicb.2025.1518275

**Published:** 2025-04-03

**Authors:** Ren Lin, Jingyi Wu, Qifa Liu

**Affiliations:** ^1^Department of Hematology, Nanfang Hospital, Southern Medical University, Clinical Medical Research Center of Hematological Diseases of Guangdong Province, Guangzhou, China; ^2^Medical Affairs, Takeda (China) International Trading Company, Shanghai, China

**Keywords:** cytomegalovirus, allogeneic hematopoietic stem cell transplantation, epidemiology, meta-analysis, China

## Abstract

**Introduction:**

Cytomegalovirus (CMV) infection poses a significant threat to individuals undergoing allogeneic hematopoietic stem cell transplantation (allo-HSCT), potentially resulting in substantial morbidity and mortality. This review summarized the epidemiology, clinical outcomes, and treatment patterns of CMV infection among allo-HSCT recipients in China.

**Methods:**

PubMed, EMBASE, the Cochrane Library, Web of Science, China National Knowledge Infrastructure (CNKI), Wanfang and Chinese Biomedical Literature Database (CBM) were systematically searched from 2013 to March 2023. All analyses were performed using R 4.1.1 software with a random effects model.

**Results:**

Fifty-six studies, which included 13,882 patients, were reviewed. The pooled overall incidence of CMV infection was 49.99% [95% confidence interval (CI) 43.72–56.26%]. Among post allo-HSCT recipients with CMV infection, 32.03% (95% CI 22.93–41.12%) developed refractory CMV infection. The overall incidence of CMV disease was 13.30% (95% CI 8.99–19.66%). The pooled all-cause mortality rate was 29.25% (95% CI 17.96–40.55%) and the CMV-related mortality rate was 3.46% (95% CI 1.19–5.73%). Results demonstrate that management of CMV has mainly focused on pre-emptive therapy due to the treatment-limiting toxicity of anti-CMV agents. Additionally, CMV infection is continuing to occur after the discontinuation of prophylaxis, highlighting the unmet need for a more effective treatment without treatment-limiting toxicities.

**Conclusion:**

This review underscores the urgent need for improved therapeutic strategies to effectively manage cytomegalovirus infection in allo-HSCT recipients, particularly in light of the high incidence and associated morbidity, as well as the limitations of current treatment options.

**Systematic review registration:**

https://www.crd.york.ac.uk/prospero/display_record.php?ID=CRD42024513908, identifier: CRD42024513908.

## 1 Introduction

Allogeneic hematopoietic stem cell transplantation (allo-HSCT) is a curative treatment for various hematological malignancies and disorders. Although this treatment modality offers the promise of hematopoietic system renewal, it poses unique challenges, particularly in terms of infectious complications, among which cytomegalovirus (CMV) emerges as a prominent adversary.

Globally, CMV infection is a well-documented threat in the context of allo-HSCT, influencing post-transplant outcomes (Camargo and Komanduri, 2017; Ljungman et al., 2017). Directly, CMV can initiate primary or reactive infection within recipients, leading to CMV viremia (Dziedzic et al., 2017). This uncontrolled viral replication may progress to CMV diseases, presenting as complications such as pneumonia, gastrointestinal complications and retinitis (Paris et al., 2009; Solano et al., 2013), contributing significantly to post-transplant morbidity and mortality (Mori and Kato, 2010; Giménez et al., 2019; Teira et al., 2016). Indirectly, CMV impedes the immune reconstitution process initiated by allo-HSCT, compromising the functionality of immune cells, particularly T cells. This weakened immune response heightens vulnerability to various opportunistic infections (Anderson-Smits et al., 2020; Nichols et al., 2002). Additionally, CMV is associated with an elevated risk of graft-vs.-host disease (GVHD), patient survival, non-relapse mortality and graft failure (Grigoleit et al., 2009; Teira et al., 2016; Fan et al., 2022; Boeckh and Geballe, 2011; Cantoni et al., 2010).

Since potent antivirals have been developed, CMV-related mortality and the incidence of CMV diseases have decreased (Gagelmann et al., 2018; Chen et al., 2018; Marty et al., 2019; Boeckh et al., 2015). Typically, management of CMV consists of prophylaxis or pre-emptive therapy. Unlike prophylactic therapy, which involves administering antiviral drugs to all at-risk patients regardless of their CMV status, pre-emptive therapy focuses on closely monitoring patients for early signs of CMV replication (e.g., through regular PCR testing) and initiating antiviral treatment only when a predefined viral load threshold is reached. Commonly employed antiviral agents for managing CMV worldwide encompass intravenous foscarnet, cidofovir, Ganciclovir and its valyl ester prodrug (valganciclovir), oral valganciclovir (Cho et al., 2019; Limaye et al., 2020). However, these drugs are associated with significant toxicity, including myelosuppression and nephrotoxicity (De Clercq and Li, 2016; George et al., 2010). Therefore, pre-emptive therapy is preferred to prophylaxis therapy in clinical practice to avoid the potential risk of drug-related adverse effects.

With CMV seroprevalence exceeding 90% in China's general population (Stem Cell Application Group, Chinese Society of Hematology, Chinese Medical Association, 2022), and haploidentical hematopoietic stem cell transplantation (haplo-HSCT) making up 60.1% of allo-HSCT cases in China (Xu et al., 2021), the likelihood of CMV infection following allo-HSCT in China is high, underlining the significant concern in this context. Therefore, this review aims to comprehensively summarize the epidemiology, clinical outcomes and treatment patterns of CMV infection among allo-HSCT recipients in China, including mainland China, Taiwan, Hong Kong and Macau.

## 2 Methods

This study followed the Preferred Reporting Items for Systematic Reviews and Meta-Analyses (PRISMA 2020) (Page et al., 2021) extension for scoping reviews (Tricco et al., 2018). The protocol was registered on PROSPERO (registration number: CRD42024513908).

### 2.1 Data sources and literature search

An electronic database search of PubMed, EMBASE, the Cochrane Library, Web of Science, CNKI, Wanfang, and CBM was conducted in March 2023 to search for studies published over the past 10 years (from 2013 to 2023) using the keywords “cytomegalovirus”, “stem cell transplantation”, and “China”. We applied no restrictions on language or publication status. The search strategy is provided in Supplementary material.

### 2.2 Study selection

Studies with standard of care that investigated allo-HSCT recipients in China were included. The following outcomes were of interest:

- Epidemiology outcomes including incidence of CMV infection, breakthrough infection which is defined as the development of an CMV infection that occurs during antiviral prophylaxis (Ljungman et al., 2019), and time to CMV infection onset;
- Clinical outcomes among patients with CMV infection including incidence of resistant CMV infection, refractory CMV infection, and recurrent CMV infection; time to CMV disease onset; and incidence of CMV disease; mortality rate; and incidence of other comorbidities with CMV infection among patients with CMV infection;
- Treatment patterns;
- Treatment-limiting toxicity.

We considered observational and interventional studies that reported in English or Chinese while excluding case reports, reviews, comments, and editorials. We excluded reports that not published in English or Chinese. Two reviewers independently screened articles for eligibility. Any disagreements were resolved through discussion with a third reviewer.

### 2.3 Data extraction, synthesis, and analysis

Two reviewers independently extracted the following data from each study into a predefined data extraction form: study characteristics including first author's name, publication year, study design, geographic location of study, and sample size; participant characteristics including age, gender, type of transplant, and population description reported by the study, as well as outcomes. Any disagreements were resolved by discussion with a third reviewer.

Meta-analyses were performed using a random effects model with the *meta* package in R (version 4.1.1) when data was appropriate. For dichotomous outcomes, we extracted the reported rates and calculated as proportion and 95% confidence interval (95% CI) for each study, then pooled them by meta-analysis. When data was insufficient for meta-analysis, we described the outcomes narratively accompanied by tabulated and charted results. We used a raincloud plot to present the time to CMV infection, CMV disease onset and CMV viremia resolution when data was available from three or more studies. In a raincloud plot, each dot represents an individual study's effect estimate, the x-axis represents the measure of effects (i.e., time in days), and the box plot within each group summarizes the distribution of these estimates, with the central line indicating the median and the box showing the interquartile range. We used a pie chart to present the proportion of various agents in different therapeutic approaches. Heterogeneity of studies was assessed by the Cochrane *Q*-test with a significance level of 0.05 and the *I*^2^ statistic, where *I*^2^ ≥ 50% coupled with *p* < 0.05 from the *Q*-test was interpreted as evidence of substantial levels of heterogeneity. We conducted subgroup analyses to explore the potential sources of substantial heterogeneity. A funnel plot was performed for outcomes reported by 10 or more studies to assess publication bias.

Subgroup analyses were carried out on the incidence of CMV infection according to days after transplantation (within 100 days and within 200 days), prophylaxis therapy (breakthrough incidence, defined as the occurrence of CMV infection during prophylaxis therapy), patient age (adults and children), transplant type (human leukocyte antigen (HLA)-matched, HLA haploidentical, cord blood and unrelated-matched), and CMV serologic status prior to transplantation [donor-negative (D–)/recipient-negative (R–), donor-positive (D+)/recipient-positive (R+), D–/R+ and D+/R–].

### 2.4 Quality assessment

The quality of included studies was assessed independently by two reviewers using Joanna Briggs Institute (JBI) critical appraisal tools for prevalence studies, case-control studies, cohort studies and randomized controlled trials (RCTs) (Barker et al., 2023; JBI, 2020). Any disagreements were resolved by discussion with a third reviewer.

## 3 Results

### 3.1 Results of study selection

**Figure 1:**
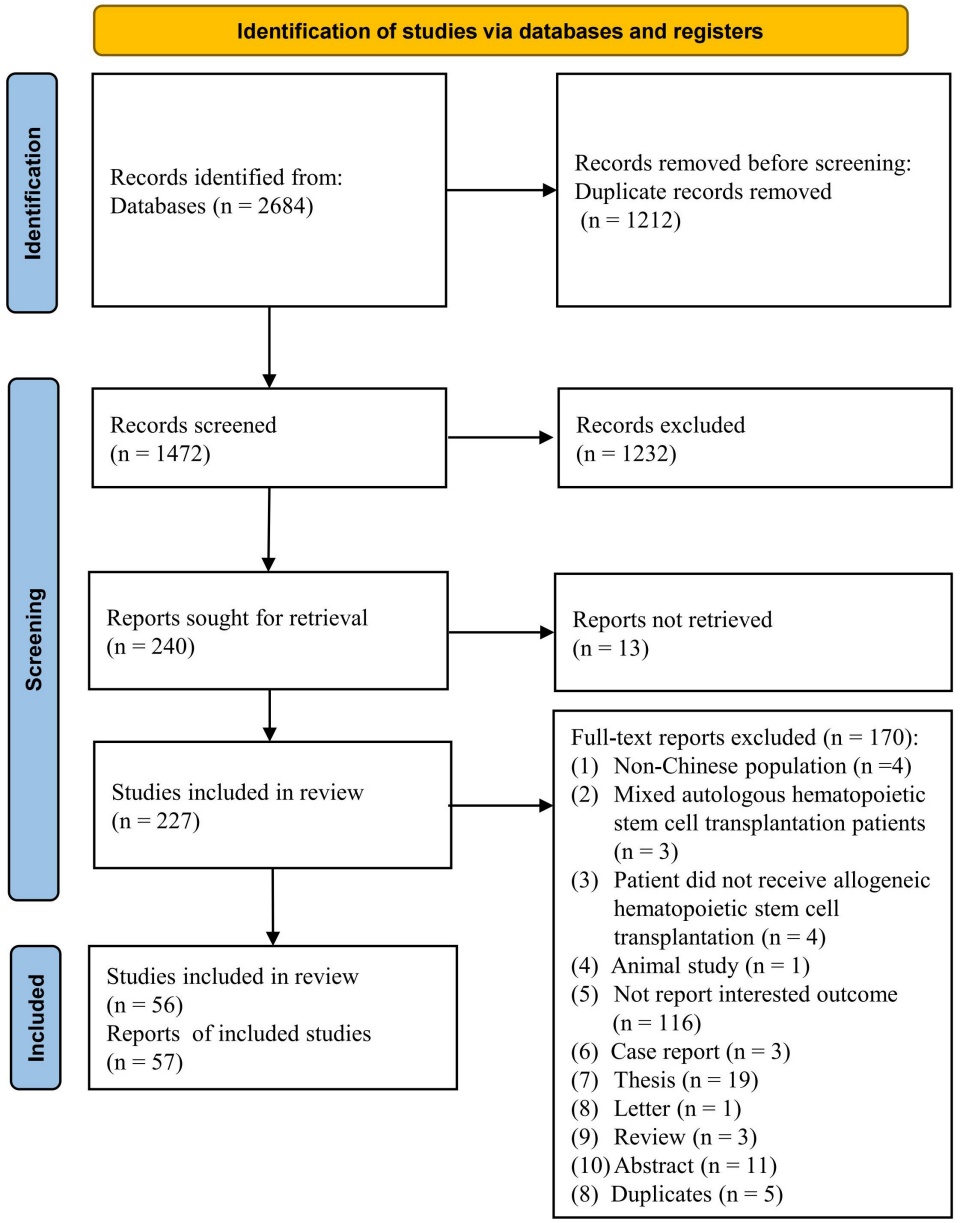
PRISMA flow chart of study selection.

The database search returned 2,684 results and provided 1,472 unique citations after duplicates were removed. A further 1,232 articles were excluded at the title- and abstract-screening stage. Full papers were sought for the remaining 240 articles. Of these, 227 full papers were retrieved and assessed for eligibility according to the inclusion and exclusion criteria. The remaining 13 articles were not successfully retrieved due to unauthorized access. Finally, 56 studies that published in 57 reports (Cao et al., 2016a,b; Gao et al., 2013; Guo et al., 2016; Han et al., 2014; He et al., 2014; Huang et al., 2022, 2013; Jin et al., 2014; Li et al., 2015a,b, 2022a, 2020, 2013b; Ma et al., 2023b; Que et al., 2014; Shi et al., 2019; Si et al., 2013; Tan et al., 2020; Wan et al., 2017; Wang et al., 2015, 2017, 2019, 2021, 2013; Wei et al., 2022; Wu et al., 2019, 2017; Xiong et al., 2023; Xu et al., 2015; Xue et al., 2019a,b; Yin et al., 2020; Zhang et al., 2016, 2014; Zhao et al., 2020; Zhao and Sun, 2021; Zhu et al., 2020; Zou et al., 2016; Bao et al., 2016; Chen et al., 2022; Cheng et al., 2022; Ding et al., 2021; Li et al., 2021, 2022b, 2013a; Lin et al., 2017; Liu et al., 2015; Meng et al., 2020; Pei et al., 2022; Tong et al., 2013; Yan et al., 2020; Yeh et al., 2022; Yin et al., 2022; Zhang et al., 2021, 2022, 2017) met the full eligibility criteria and were included in the review. The study selection process is shown in Figure 1.

### 3.2 Characteristics and quality of included studies

We included a total of 56 studies with sample sizes ranging from 11 to 3,862 (Table 1). These studies comprised 25 cross-sectional studies, 24 cohort studies, five case-control studies, and two RCTs. The quality assessment results of the included studies are summarized in Supplementary Tables S1–S4.

**Table 1:** Summary of characteristics of included studies.

**Study ID (first author and publication year)**	**Study design**	**Institution**	**Sample size**	**Diagnosis (number of patients)**	**Transplant type (number of patients)**	**Age (years)**	**CMV DNA in aqueous humor (copies/mL)**
Bao 2016	Cohort	The First Affiliated Hospital of Soochow University	685	AL: 495 NHL: 34 AA/PNH: 38 MDS/MPD: 68 CML: 47 Other: 3	Related HLA-identical sibling: 237 Related HLA-haploidentical: 261 Matched unrelated: 187	Median (range): 31 (2–58)	NR
Cao 2016	Cross-sectional	The First Affiliated Hospital of Zhengzhou University	134	AML: 43 CML: 53 ALL: 14 AA: 15 Others: 9	Haploid: 69 Full compatibility of siblings: 60 Non-related compatibility: 5 Peripheral hematopoietic stem cell transplantation: 121 Bone marrow combined with peripheral blood transplantation: 13	Median (range): 23 (5–50)	Median: 3.95 × 10^3^
Chen 2022	Case control	National Clinical Research Center for Blood Diseases, Haihe Laboratory of Cell Ecosystem, Institute of Hematology and Blood Diseases Hospital	359	AML: 237 ALL: 112 MAL: 10	Haploidentical-related donor: 195 MSD: 164	NR	NR
Cheng 2022	Cohort	National Cheng Kung University Hospital	11	ALL: 5 AML: 3 SAA: 2 HSTCL: 1	Matched sibling: 2 Matched unrelated: 5 Mismatched unrelated: 4	Median (range): Letermovir group: 16.1 (9.2–17.8) Control group: 13.9 (4.5–16.9)	NR
Ding 2021	Cross-sectional	The First Affiliated Hospital of Soochow University	160	NHL: 160	HLA-matched donor: 66 HLA-mismatched donor: 94	Median (range): 30 (5–59)	NR
Gao 2013	Cross-sectional	Anhui Medical University Provincial Hospital	107	AML: 41 ALL: 48 CML: 10 MDS: 4 NHL: 3 MPL: 1	Unrelated cord blood HSCT: 107	Median (range): 15 (2–46)	NR
Guo 2016	Cross-sectional	Beijing Military General Hospital	50	AML: 20 ALL: 12 AAA: 10 MDS: 6 Lymphoma: 2	Haplo-HSCT: 50	Mean (range): 23.8 (5–45)	Range: 2.4 × 10^3^ – 6.2 × 10^6^
Han 2014	Cross-sectional	First Affiliated Hospital of Anhui Medical University	82	CML: 31 AML: 20 AA: 15 ALL: 16	Allogeneic peripheral blood stem cell transplantation: 16 Allogeneic bone marrow transplantation: 13 Umbilical cord blood stem cell transplantation: 10 Allogeneic bone marrow + peripheral blood stem cell transplantation: 43 HLA fully matched: 73 HLA partially matched: 9	Age: no. of patients <14: 1 15–45: 58 ≥45: 23	NR
He 2014	Cross-sectional	Zhongda Hospital Affiliated to Southeast University School of Medicine	108	AML: 24 ALL: 19 CML: 30 MDS: 20 LP: 4 SAA: 11	Completely matched unrelated donors: 26 Unrelated donor haploidentical: 17 Completely matched related donors: 44 Related donor haploidentical: 21	Age: no. of patients ≤ 18: 21 18–40: 43 ≥40: 44	NR
Huang 2013	Cross-sectional	Peking University People's Hospital Institute of Hematology	327	NR	Sibling fully matched transplants: 96 Haploidentical transplants from relatives: 216	Median (range): 29 (2–67)	Median: 1.1 × 10^4^
Huang 2022	Case control	Guangdong Women and Children Medical Center Hematology and Oncology Department	257	Severe beta thalassemia: 257	HLA fully matched donors: 195 (106 related/89 unrelated) HLA partially matched donors: 65 (27 related/38 unrelated)	Median (IQR): 6 (4–8)	NR
Jin 2014	Cross-sectional	The Affiliated Hospital of the Academy of Military Medical Sciences	115	AML: 31 ALL: 25 CML: 42 AA: 4 NHL: 5 MDS: 7 CLL: 1	Allogeneic peripheral blood HSCT recipients: 102 (non-myeloablative transplantation: 8 and haploidentical transplantation: 5) Allogeneic bone marrow HSCT recipients: 9 Allogeneic bone marrow + peripheral blood HSCT recipients: 4	Mean (range): 34 (17–58)	Median: 4.0 × 10^3^
Kong 2015	Cohort	The Peking University People's Hospital	488	AML: 203 ALL: 158 MDS: 60 CML: 26 SAA: 26 FA: 1 Lymphoma: 9 Myeloma: 4 Myelofibrosis: 1	Matched sibling: 91 Mismatched family: 397	Median (range): 29 (2–61)	NR
Li 2013a	Cohort	Chinese PLA General Hospital	141	AML: 57 ALL: 24 NHL: 16 MDS: 12 AA: 10 Chronic myelogenous leukemia: 19 Chronic myelomonocytic leukemia: 1 Chronic lymphocytic leukemia: 1 Other: 1	HLA-matched unrelated: 43 HLA-matched related: 79 HLA-mismatched related: 19	Median (range): 32 (10–56)	Mean (95%CI): 1.53 × 10^3^ (95% CI 1.39 × 10^3^ – 1.81 × 10^3^)
Li 2013b	Cohort	Affiliated Hospital of the Academy of Military Medical Sciences	63	AML: 15 ALL: 13 CML: 22 AA: 4 NHL: 4 MDS: 4 CLL: 1	Allogeneic peripheral blood HSCT recipients: 51 (including non-myeloablative transplantation: 4 and haploidentical transplantation: 2) Allogeneic bone marrow HSCT recipients: 7 Allogeneic bone marrow + peripheral blood HSCT recipients: 4 Umbilical cord blood HSCT recipient: 1	Mean (range): 33 (16–56)	NR
Li 2015a	Cross-sectional	Anhui Medical University Provincial Hospital	100	CML: 5 ALL: 57 AML: 31 AMLL: 1 MDS: 4 Malignant lymphoma: 2	Unrelative cord blood transplantation: 100	Median (range): 13 (1.5–51)	NR
Li 2015b	Cohort	Hematology Department, The Second Affiliated Hospital of Chongqing Medical University	30	AML: 12 CML: 7 CLL: 3 ALL: 3 SAA: 2 MDS transit to AML-M: 2 NHL: 1	Haplo-HSCT: 13 Unrelated HLA fully matched HSCT: 6 HLA-matched sibling HSCT: 11 (Among them: 10 cases involved peripheral blood stem cell transplantation, 5 cases involved a combination of peripheral blood stem cells and bone marrow transplantation, 15 cases involved a combination of peripheral blood stem cells, bone marrow, and umbilical cord blood mesenchymal stem cell transplantation)	Median (range): 42 (12–60)	NR
Li 2020	Cross-sectional	Hematology Department, Shanghai First People's Hospital	411	AML: 14 ALL: 10 MDS: 5 NHL: 3 AA: 2 (34 cases with CMV pneumonia)	Haploid: 230 HLA-matched related donors: 103 Unrelated donors: 78	Median (range): 32 (8–62) (34 cases of CMV pneumonia)	Median (range): 1.45 × 10^5^ (1.1 × 10^4^ – 1.10 × 10^8^)
Li 2021	Cohort	Division of Hematology/Medical Oncology, Department of Medicine, Taichung Veterans General Hospital	84	AML: 58 ALL: 26	Haploid: 27 Haplotype match with a sibling: 37 Unrelated donor full match: 20	Median (range): 43 (18–74)	NR
Li 2022a	Cross-sectional	Hematology Department, Peking University People's Hospital Institute	327	AML: 154 ALL:102 MDS: 39 Others: 32	Haploid: 241 Compatible siblings: 74 All unrelated donors are identical: 12	Median (range): FSC/GCV: 34 (14-64) CDV (first line): 32 (27–53) CDV (second line): 37 (16–57)	Median (Q1, Q3): Co-reactivation:1.71 × 10^3^ (1.14 × 10^3^, 3.68 × 10^3^); CMV reactivation:1.69 × 10^3^(0.77 × 10^3^, 3.50 × 103)
Li 2022b	Case control	Hematology Department, Ruijin Hospital Affiliated to Shanghai Jiao Tong University School of Medicine	408	AML: 146 CML: 40 ALL: 163 MDS: 37 AA: 22	Cord blood: 152 Peripheral blood stem cells: 92 Bone marrow: 48 Peripheral blood stem cells + bone marrow: 116	Age: no. of patients <40: 306 ≥40: 102	NR
Lin 2016	Cross-sectional	Taichung Veterans General Hospital	82	AML: 39 ALL: 20 MDS: 1 CML: 3 AA: 9 Multiple myeloma: 2 Lymphoma: 8	Matched sibling donor: 27 Matched unrelated: 21 Mismatched unrelated: 29 Haploidentical: 5	Mean ± SD: 41.98 ± 14.57	NR
Ma 2023	Cohort	Peking University Institute of Hematology	335	Single-drug therapy group: AML: 59 ALL: 31 MDS: 33 NHL: 10 AA: 14 Others: 9 Combination therapy group: AML: 24 ALL: 14 MDS: 10 NHL: 3 AA: 7 Others: 7	Haplo-HSCT: 335	Median (range): Single-drug therapy group: 29 (1–66) Combination therapy group: 32 (1-65)	Median: Single-drug group: 5.4 × 10^3^; Combination-drug group: 4.5 × 10^3^
Meng 2020	Cross-sectional	Peking University People's Hospital	3,862	NR	Haploid: 3,862	NR	NR
Pei 2022	Cohort	Peking University People's Hospital	190	AML: 84 ALL: 73 MDS: 16 Others: 17	Haploid: 39	Median (range): 29 (2–66)	NR
Que 2014	Cross-sectional	Hematology and Oncology, Children's Hospital Affiliated to Chongqing Medical University	26	PID: 26	Sibling bone marrow transplant: 4 Cord blood transplant: 22	Median (range): 27 (7–77) months	NR
Shi 2019	Cross-sectional	Peking University People's Hospital	61	AML: 28 ALL: 25 AHL: 2 MDS: 6	Haploid: 39	Median (range): 30 (16–56)	NR
Si 2013	Cohort	Affiliated Bayi Children's Hospital, Beijing Military General Hospital	47	AML: 11 SAA: 8 High-risk ALL: 7 HPS: 6 DBA: 5 AMLL: 5 MDS: 2 NHL: 2 OF: 1	Haploid: 24 Sibling donor transplants: 16 Unrelative donor transplants: 7	Mean ± SD (range): 8.0 ± 6.4 (0.3–13.2)	NR
Tan 2020	RCT	Army Medical University Xinqiao Hospital Hematology Medical Center	69	NR	HLA fully matched: 28 HLA haploidentical matched: 41	Median (range): 32 (4–59)	Median (range): Ganciclovir group: 1.69 × 10^3^ (5.66 × 10^2^ – 1.83 × 10^3^); Foscarnet group: 2.06 × 10^3^ (4.46 × 10^2^ – 2.09 × 10^4^)
Tong 2013	Cohort	Anhui Provincial Hospital (an affiliate of Anhui Medical University), Shandong Second Hospital (an affiliate of Shandong University School of Medicine) or Wuhu Yijishan Hospital (an affiliate of Wannan Medical College)	176	Hematological malignancies: 176	Umbilical cord blood transplantation from unrelated donors: 176	Mean (range): 20 (2.2–51)	NR
Wan 2017	Cross-sectional	Affiliated Hospital of Academy of Military Medical Sciences	41	SAA: 41	AHSCT: 41	Median (range): 26 (9–54)	NR
Wang 2013	Cohort	First Affiliated Hospital, Xi'an Jiaotong University School of Medicine	37	AML: 18 (among them: M1: 2, M2a: 11, M2b: 1, M4a: 1, M4b: 2, M5b: 1) ALL-L1: 4 ALL-L: 28 CML: 6 MPAL: 1	Allogeneic peripheral blood HSCT: 36 Bone marrow transplantation: 1 Unallogeneic HSCT: 16 Related HSCT: 21 HLA partially matched: 11 HLA fully matched transplantation: 26	Median (range): 29 (11–59)	NR
Wang 2015	Cohort	Tri-Service General Hospital Hematopoietic Stem Cell Transplantation Center	83	AML: 29 ALL: 17 MDS: 2 SAA: 32 CML-CP: 2 BP: 1	HSCT-HLA-matched (URD and MSD): 33 Haploid: 50	Median (range): 15 (2–48)	NR
Wang 2017	Cross-sectional	Pediatrics Department, Nanfang Hospital, Southern Medical University	310	NR	Fully HLA-matched transplantation: 217 Half-matched transplantation: 93	Median (range): 5 (3–16)	NR
Wang 2019	Cross-sectional	Beijing Kyoto Children's Hospital Hematology and Oncology Department	167	NR	Haplo-HSCT: 167	Mean ± SD: 6.96 ± 3.98	NR
Wang 2021	Cross-sectional	Guangzhou First Municipal People's Hospital	270	SAA: 270	Haploid: 94 Compatible siblings: 108 Unrelated complete compatibility: 68	Median (range): 7 (1–14)	NR
Wei 2022	Case control	Children's Hospital of Fudan University	Total: 143 No CMV infection: 112 CMV infection: 31	PID: 143	Cord blood: 143	Median (range): 33 (25–44)	Median (Q1, Q3): 32.8 × 10^3^ (18.3 × 10^3^, 63.1 × 10^3^)
Wu 2017	Cross-sectional	The First Affiliated Hospital of PLA General Hospital	96	AML: 31 ALL: 23 MDS: 5 NHL: 9 SAA: 28	HLA-half-matched: 73 HLA-matched: 23	NR	Median (range): 3.12 × 10^3^ (5.10 × 10^2^ – 6.30 × 10^5^)
Wu 2019	Cross-sectional	Guangdong Provincial People's Hospital	53	AML: 30 Lymphoma: 17 MDS: 1 MPN: 1 CML: 2 Myeloid sarcoma: 2	Unrelated: 17 Related: 36 Primary source of stem cells from peripheral blood: 45 Bone marrow: 5 Cord blood: 3	Median (range): 30 (16–54)	NR
Xiong 2023	Cross-sectional	First Affiliated Hospital of Chongqing Medical University	246	AML: 108 ALL: 60 AMLL: 6 MDS: 18 CML: 7 SAA: 28 MPN: 1 PNH: 1 Multiple myeloma: 7 Lymphoma: 10	Haplo-HSCT: 167 Sibling fully matched transplantation: 66 Unrelated donor transplantation: 13 (5 cases with HLA full match, 8 cases with 9/10 match)	Median (range): 32 (14–65)	NR
Xu 2015	Cohort	Peking University People's Hospital Hematology Research Institute	28	AML: 10 ALL: 8 MDS: 4 CML: 3 AA: 2 CGD: 1	Haploid: 25 Haplotype match with a sibling: 2 Unrelated cord blood transplant: 1	Median (range): 29.5 (3–57)	Median (range): 3.10 × 10^3^ (0.49–46.50)
Xue 2019a	Cohort	North China University of Science and Technology Affiliated Hospital	60	AML: 24 ALL: 20 CML: 6 MDS: 10	HLA-matched: 22 HLA-mismatched: 38	NR	NR
Xue 2019b	Cohort	Huabei University of Science and Technology Affiliated Hospital	40	AML: 12 ALL: 9 MDS: 5 CML: 3 AA: 11	HLA fully matched: 15 HLA partially matched: 25	NR	NR
Yan 2020	Cross-sectional	Peking University Institute of Hematology	1,466	AL or MDS: 1,466	Haplo-HSCT: 1,446	Median (range): 28 (1–66)	Median (range): 1.56 × 10^5^ (6.74 × 10^3^ – 3.26 × 10^6^)
Yeh 2022	Cross-sectional	Kaohsiung Medical University Hospital	180	AML: 85 ALL: 48 CML: 10 SAA: 15 MDS: 15 NHL: 7	NR	Median ± SD: 39.22 ± 11.76	NR
Yin 2020	Cohort	Nanfang Hospital, Southern Medical University	17	AML: 10 NHL: 1 ALL: 3 MDS: 3	Haploid: 17	Median (range): 30 (16–56)	Median (range): 2.662 × 10^3^ (0.388 × 10^3^ – 4.61 × 10^4^)
Yin 2021	Cohort	Nanfang Hospital, Southern Medical University	141	AML: FSC/GCV: 59, CDV: 5 ALL: FSC/GCV: 26, CDV: 2 MDS: FSC/GCV: 14, CDV: 2 NHL: FSC/GCV: 1, CDV: 0 MPAL: FSC/GCV: 1, CDV: 0	Haploid: 141	Median (range): 28 (5.5–58)	Median (range): 8.65 × 10^3^ (0.894 × 10^3^ – 4.91 × 10^5^)
Zhang 2014	Cross-sectional	The 307th Hospital of the Chinese People's Liberation Army	80	AML: 30 CML: 9 ALL: 23 SAA: 11 MDS: 7	HLA-matched: 53 HLA-half-matched: 27	Median (range): 33 (8–58)	NR
Zhang 2016	Cohort	Third Military Medical University; Xinqiao Hospital; National Center for Hematological Diseases	165	AML: 71 ALL: 24 CML: 23 SAA: 2 Lymphoma/ lymphocytic leukemia: 13 AMLL: 5 MDS: 3 PNH: 1 MA: 1	Haploid: 99 HLA fully matched among relatives: 50 Unrelated full-matched: 16	Median (range): 26 (2–54)	NR
Zhang 2017	Cohort	Guangzhou First People's Hospital	12	SAA: 10 AML: 10 AHL: 1	HLA-related: 3 HLA-unrelated: 4 HLA-mismatched donors: 5	Mean ± SD (range): 26.6 ± 9 (18–49)	NR
Zhang 2021	Cohort	Hematology & Hospital of Blood Diseases, Chinese Academy of Medical Sciences	73	ALL: 11 AML: 39 MDS: 13 SAA: 4 Others: 6	Haplotype: 61 Compatible siblings: 12	Age: no. of patients ≤40: 38 >40: 35	Median (range): 1.73 × 10^3^ (1.001 × 10^3^ – 9.2278 × 10^4^)
Zhang 2022	Cohort	Guangzhou First People's Hospital	155	SAA: 155	Allogeneic HSCT (MSD): 71 Alternative donor (HID and URD): 84	Mean ± SD: 29.14 ± 9.12	9.77 × 10^3^ ± 1.27 × 10^4^
Zhao 2020	Cohort	Peking University Institute of Hematology	122	AML: 47 ALL: 50 MDS: 15 CML: 10	Haplo-HSCT: 122	Median (range): 29 (1–63)	NR
Zhao 2021	RCT	Henan University of Science and Technology First Affiliated Hospital	78	NR	NR	Median (range): Total: 14 (8–27) months No CMV infection: 13.5 (7.3–26.0) months CMV infection: 15.0 (10.0–32.0) months	NR
Zhu 2020	Case control	Affiliated Children's Hospital of Soochow University	269	AL: 148 MDS/MPN: 18 Bone marrow failure diseases: 61 Immunodeficiency diseases: 26 Others: 16 [7 cases of MA; 1 case of NHL (IV); 1 case of chronic EB virus infection; 1 case of Crisp Oni syndrome; 3 cases of adrenoleukodystrophy; 2 cases of mucopolysaccharidosis type II; 1 case of pyruvate kinase deficiency]	HLA-matched sibling donors: 33 HLA-haploidentical sibling donors: 116 Unrelated donors: 120	Median (range): 65 (33–115) months	Median (range): RCI group:5.78 × 10^4^ (2.1 × 10^4^ – 1.5125 × 10^5^); non-RCI group: 8.31 × 10^3^ (2.845 × 10^3^ – 2.315 × 10^4^)
Zou 2016	Cohort	Affiliated Hospital of the Academy of Military Medical Sciences	398	AML: 195 ALL: 141 MDS: 38 AA: 7 CML: 11 Others: 6	Unrelative allogeneic HSCT: 164 High-resolution matched sibling allogeneic HSCT: 165 Haplo-HSCT: 69	Median (range): 33 (10–64)	NR

AA, aplastic anemia; AAA, acute aplastic anemia; AHL, acute hybrid leukemia; AHSCT, allogeneic hematopoietic stem cell transplantation; AL, acute leukemia; ALL, acute lymphoid leukemia; AML, acute myelocytic leukemia; AMLL, acute mixed-lineage leukemia; AP, accelerated phase; BAL, biphenotypic acute leukemia; BC, blast crisis; CDV, cidofovir; CGD, chronic granulomatous disease; CML, chronic myeloid leukemia; CML-CP, chronic myelogenous leukemia in chronic phase; CMV, cytomegalovirus; DBA, Diamond-Blackfan anemia; EB, Epstein-Barr; FA, Fanconi anemia; FSC, foscarnet; GCV, ganciclovir; Haplo-HSCT, haploidentical hematopoietic stem cell transplantation; HID, haploidentical donor; HLA, human leukocyte antigens; HPS, hemophagocytic syndrome; HSTCL, hepatosplenic T-cell lymphoma; IV, intravenous; MA, Mediterranean anemia; MDS, myelodysplastic syndrome; MPAL, mixed-phenotype acute leukemia; MPD, myeloproliferative disorders; MPL, mixed-phenotype leukemia; MPN, myeloproliferative neoplasm; NHL, non-Hodgkin lymphoma; NR, not reported; OF, osteopetrosis; PID, primary immunodeficiency diseases; PNH, paroxysmal nocturnal haemoglobinuria; SAA, severe aplastic anemia; SD, standard deviation; URD, unrelated donor.

### 3.3 Epidemiology outcomes

#### 3.3.1 Incidence of CMV infection and time to CMV infection onset

**Figure 2:**
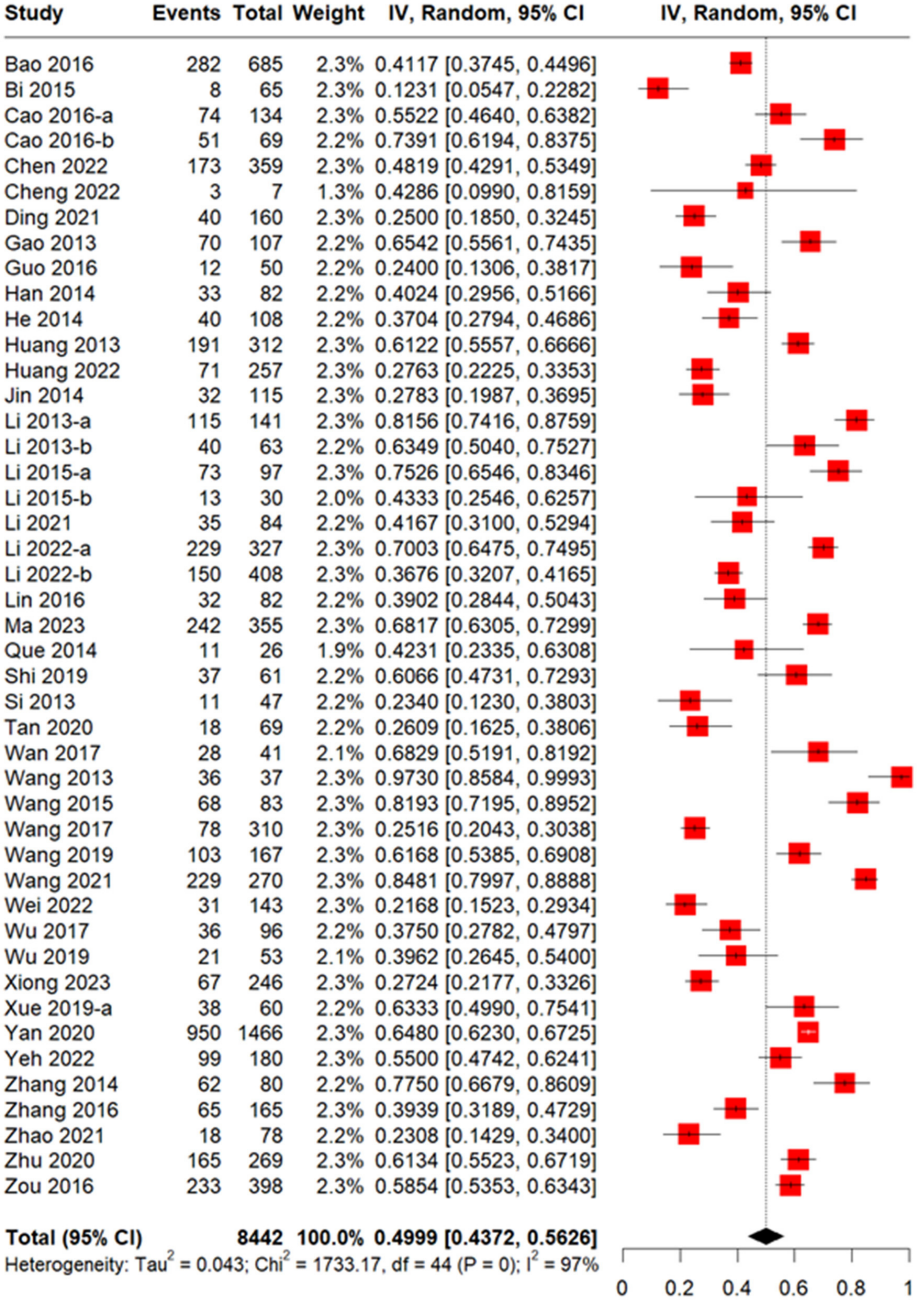
Forest plot of incidence of CMV infection in allogeneic hematopoietic stem cell transplantation (allo-HSCT) recipients.

**Figure 3:**
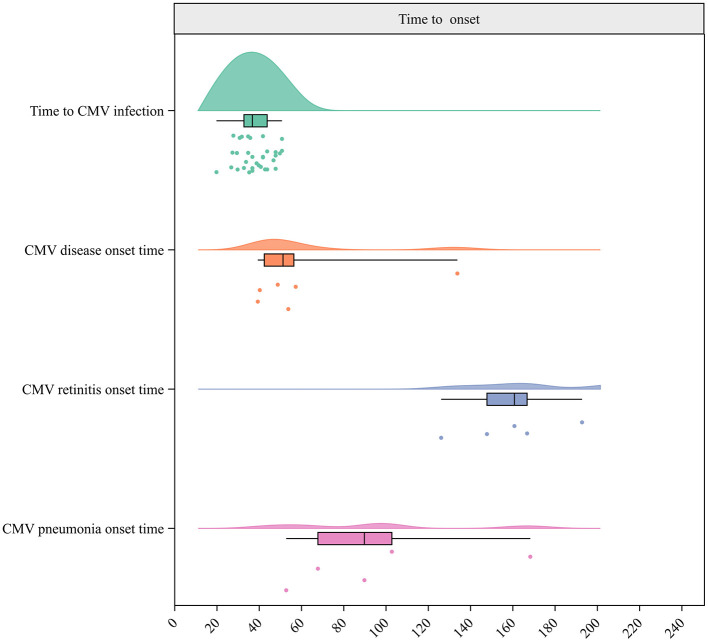
Raincloud plot for times to CMV infection, CMV breakthrough infection, CMV disease, CMV retinitis, and CMV pneumonia onset.

The meta-analysis of 47 studies conducted on 8,442 patients revealed that the incidence of CMV infection was 49.99% (95% CI 43.72–56.26%, Figure 2). The median time for CMV infection to develop after allo-HSCT was provided in 18 studies with 3,806 patients and found to be 37 days (Figure 3). Three studies with 151 patients reported the incidence of CMV breakthrough infection during foscarnet, ganciclovir and letermovir prophylactic therapy, and the pooled result was 7.29% (95% CI 3.09–11.48%, Supplementary Figure S1).

#### 3.3.2 Incidence of CMV infection based on transplantation type

Regarding transplant types, subgroup analyses revealed that the incidence of CMV infection was 70.82% (95% CI 58.29–83.35%) in unrelated matched transplantation, 69.08% (95% CI 61.50–76.66%) in haploidentical transplantation, 55.48% (95% CI 31.29–79.67%) in cord blood transplantation and 36.15% (95% CI 22.93–49.36%) in HLA-matched transplantation (Table 2, Supplementary Figures S2–S5).

**Table 2:** Incidence of CMV infection based on days after transplantation, patient age, transplantation type, and donor and recipient CMV serologic status—subgroup analysis results.

**Subgroup analysis**	**Number of studies**	**Sample size**	**Pooled incidence (95% CI, *I*^2^)**
**Days after transplantation**			
Within 100 days	19	2654	47.46% (31.90–63.02%, 99%)
Within 200 days	4	520	67.50% (53.85–81.14%, 91%)
**Age**			
Children	9	1,291	38.92% (26.14–52.49%, 95%)
**Transplantation type**			
Unrelated matched	8	193	70.82% (58.29–83.35%, 73%)
Haploidentical	14	2,879	69.08% (61.50–76.66%, 94%)
Cord blood	5	438	55.48% (31.29–79.67%, 97%)
HLA matched	10	742	36.15% (22.93–49.36%, 93%)
**Donor and recipient CMV serologic status**			
D– and R+	6	313	57.64% (43.59–71.69%, 78%)
D– and R–	16	1061	47.99% (35.38–60.61%, 95%)
D+ and R+	5	2183	45.86% (95% CI 32.77–58.95%, 97%)
D+ and R–	6	142	34.52% (12.27–56.77%, 88%)

CI, confidence interval; CMV, cytomegalovirus; HLA, human leukocyte antigens; “+” refers to positive CMV serologic status, “–” refers to negative CMV serologic status.

#### 3.3.3 Incidence of CMV infection based on donor and recipient CMV serologic status

The pooled incidence of CMV infection was 57.64% (95% CI 43.59–71.69%) based on the CMV serologic status of D–/R+, 47.99% (95% CI 35.38–60.61%) under the status of D–/R–, 45.86% (95% CI 32.77–58.95%) under the status of D+/R+ and 34.52% (95% CI 12.27–56.77%) under the status of D+/R– (Table 2, Supplementary Figures S6–S9).

#### 3.3.4 Incidence of cumulative CMV infection based on days after transplantation

The results showed that the incidence of cumulative CMV infection was 47.46% (95% CI 31.90–63.02%) within 100 days of transplantation and 67.50% (95% CI 53.85–81.14%) within 200 days (Table 2, Supplementary Figures S10, S11).

#### 3.3.5 Incidence of CMV infection based on patient age

Two studies (Li et al., 2015a, 2021) reported incidences of CMV infection among adult patients of 70.03 and 41.67%, respectively. In addition, the pooled results revealed that the incidence of CMV infection was 38.92% (95% CI 26.14–52.49%) among pediatric patients (Table 2, Supplementary Figure S12).

### 3.4 Clinical outcomes among patients with CMV infection

#### 3.4.1 Incidence of refractory, recurrent, and resistant CMV infection

After transplantation, 32.03% (95% CI 22.93–41.12%) of patients with CMV infection developed refractory CMV infection (five studies with 787 patients) and 15.42% (95% CI 7.76–30.63%) experienced CMV recurrence (12 studies with 697 patients, Supplementary Figures S13 and S14). Only one study on resistant CMV infection, which included 143 patients, was identified. Drug resistance rate seen in 0.7%.

#### 3.4.2 Incidence of CMV disease and time to CMV disease onset

**Figure 4:**
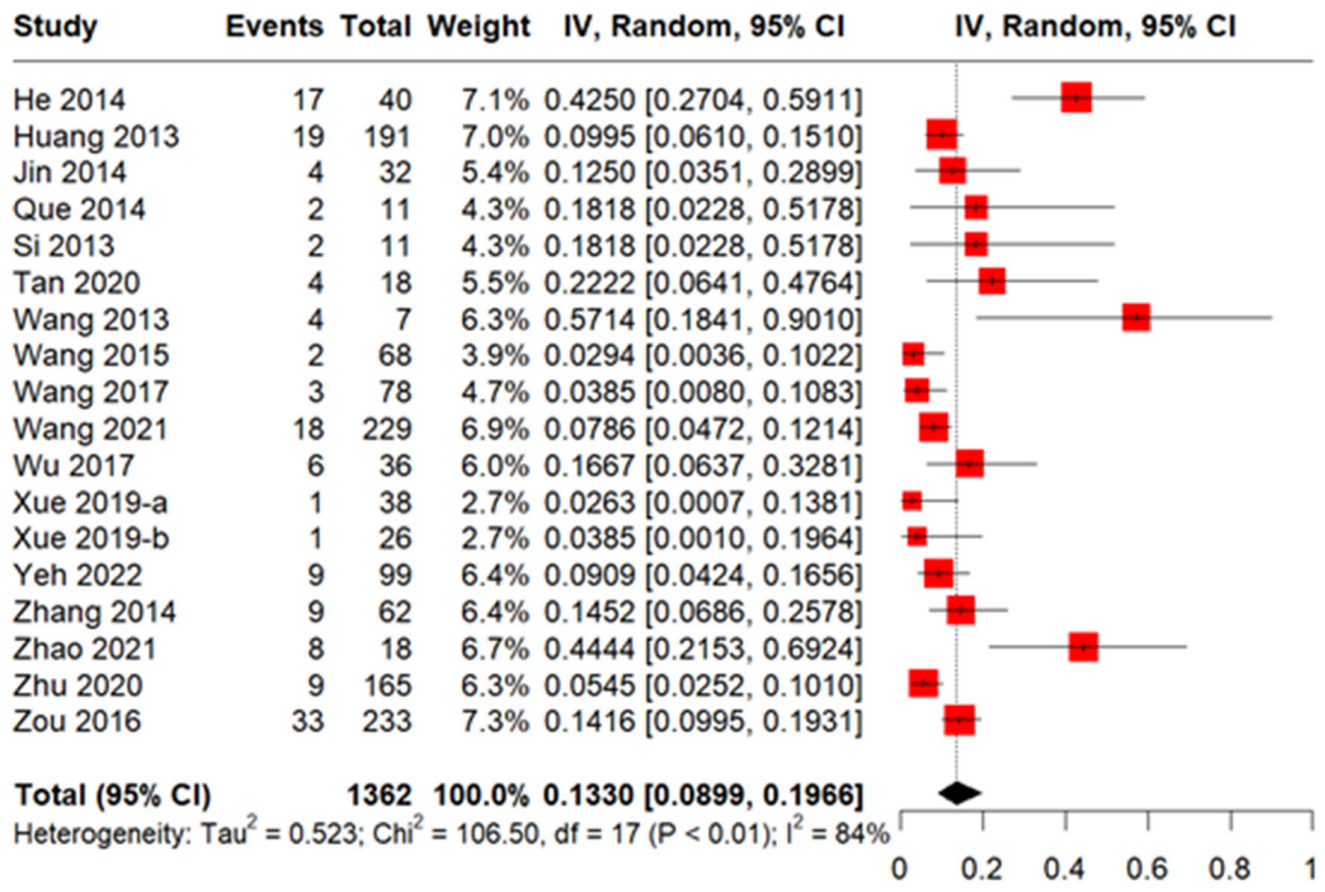
Forest plot of incidence of CMV disease in allogeneic hematopoietic stem cell transplantation (allo-HSCT) recipients with CMV infection.

A total of 18 studies including 1,362 patients reported the incidence of CMV disease among patients with CMV infection. The pooled incidence was 13.30% (95% CI 8.99–19.66%, Figure 4). The median CMV disease onset time was 51.5 days, based on six studies with 4,737 patients (Figure 3). Two studies (Ma et al., 2023b; Zhu et al., 2020) reported incidences of CMV disease among patients with refractory CMV infection of 3.4 and 3.3%, respectively.

Specifically, the incidence of CMV pneumonitis among patients with CMV infection pooled from 23 studies involving 7,434 patients was 5.96% (95% CI 4.44–8.00%, Supplementary Figure S15). The median time for CMV pneumonia onset was 90 days, based on five studies with 4,675 patients (Figure 3). Moreover, the pooled incidence was 13.48% for CMV cystitis, 7.12% for enteritis, 3.47% for retinitis, 0.59% for encephalitis, and 5% for hepatitis (He et al., 2014; Table 3, Supplementary Figures S16–S19). The median times to the onset of specific CMV diseases are shown in Table 4.

**Table 3:** Incidence of CMV disease among patients with CMV infection in China.

**CMV disease**	**Number of studies**	**Sample size**	**Pooled incidence (95% CI, *I*^2^)**
Pneumonitis	23	7434	5.96% (4.44–8.00%, 83%)
Cystitis	6	218	13.48% (4.88–22.08%, 77%)
Enteritis	8	518	7.12% (2.50–20.30%, 87%)
Retinitis	8	1,893	3.47% (1.60–5.33%, 81%)
Encephalitis	3	384	0.59% (0.00–1.35%, 0%)
Hepatitis	1	40	5.00%

CI, confidence interval; CMV, cytomegalovirus.

**Table 4:** Median times to onset of CMV diseases after transplantation.

**CMV disease**	**Number of studies**	**Sample size**	**Median time to onset**
Pneumonitis	5	4,675	90 days
Cystitis	1	165	36 days
Enteritis	2	115 and 3,862	41 and 46 days
Retinitis	5	5,662	161 days
Encephalitis	1	3,862	85 days

#### 3.4.3 Mortality rate

The all-cause mortality rate was reported in nine studies with 710 patients and was found to be 29.25% (95% CI 17.96–40.55%, Supplementary Figure S20), whereas the CMV-related mortality rate was found to be 3.46% (95% CI 1.19–5.73%), based on 14 studies with 632 patients (Supplementary Figure S21).

#### 3.4.4 Incidence of comorbidities

Results pooled from four studies with 283 patients indicated that 64.20% (95% CI 31.22–97.18%) of patients with CMV infection experienced GVHD (Supplementary Figure S22). Another four studies with 248 patients reported an incidence of acute GVHD of 63.97% (95% CI 17.53–100.00%, Supplementary Figure S23). Two studies (Tong et al., 2013; Huang et al., 2022) reported incidences of chronic GVHD of 14.08% and 16.00%, and two studies (Yang et al., 2022; Huang et al., 2022) reported incidences of coexisting EB virus infection of 32.75 and 29.58%, respectively. The incidence of coexisting bacterial or fungal infection was 78.13%, as reported in one study (He et al., 2014) with 32 patients.

#### 3.4.5 Time to CMV viremia resolution

**Figure 5:**
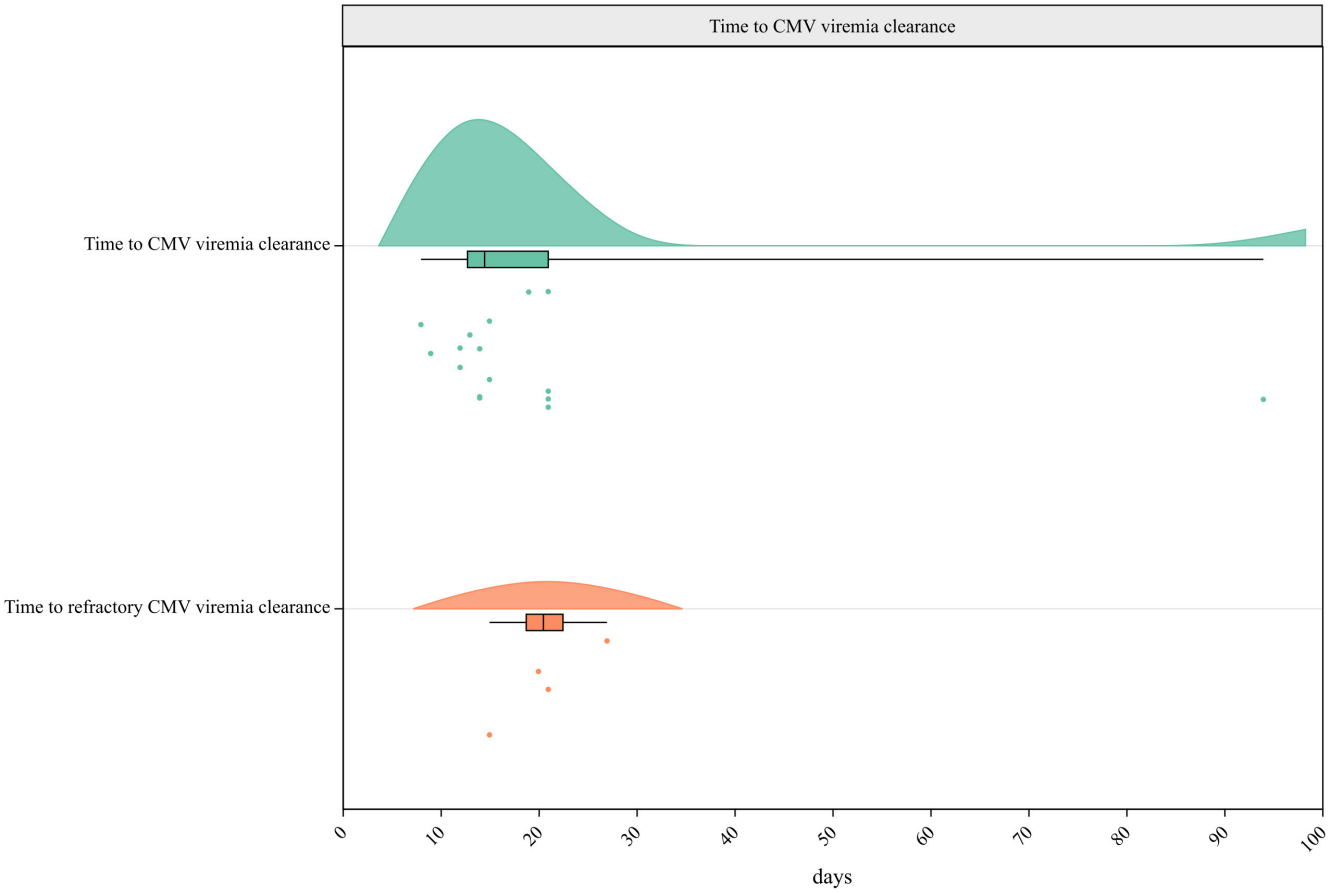
Raincloud plot for times to CMV and refractory CMV viremia resolution.

Eleven studies provided data on the time to CMV viremia resolution, and the median time was 14.5 days. The median time to refractory CMV viremia resolution, reported in three studies, was 20.5 days (Figure 5).

#### 3.4.6 Treatment patterns

**Figure 6:**
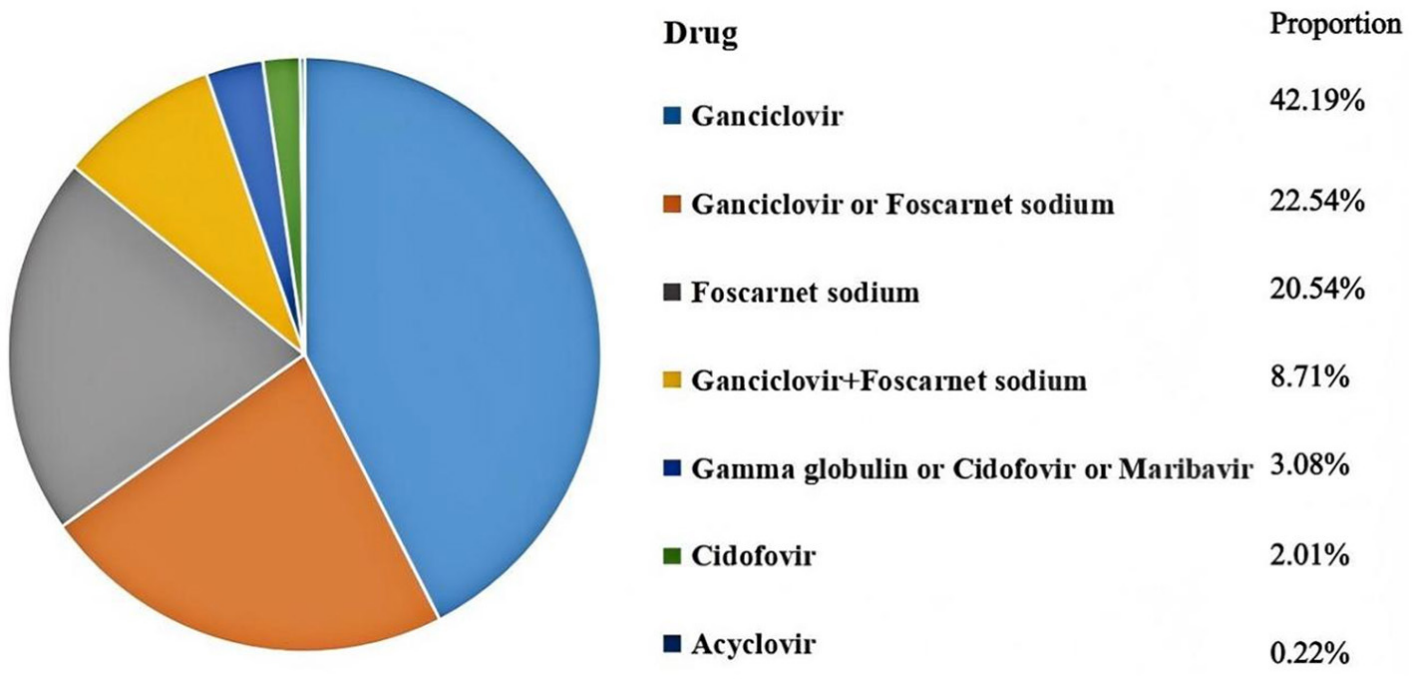
Proportion of various drugs used in pre-emptive therapy.

Pre-emptive therapy was reported in 24 studies with 5,239 patients. Results indicated that ganciclovir was the most commonly used drug (42.19%), followed by foscarnet (20.54%) (Figure 6). For prophylaxis therapy, we identified the utilization of ganciclovir, ganciclovir in combination with acyclovir, foscarnet, foscarnet in combination with acyclovir, and letermovir.

#### 3.4.7 Treatment-limiting toxicity

We identified six studies that reported treatment-related adverse events associated with the use of cidofovir, foscarnet, ganciclovir, a combination of foscarnet and acyclovir, and a combination of ganciclovir and acyclovir. According to three studies, 34.90–41.20% of patients treated with ganciclovir experienced myelosuppression. Specifically, one study (He et al., 2014) reported that 34.90% patients experienced myelosuppression, one study (Tong et al., 2013) reported that 35.30% patients experienced neutrophilic leukopenia and one study (He et al., 2014) reported that 41.20% patients experienced granulocytopenia (grade III), and one study (He et al., 2014) reported that 11.40% patients experienced impaired kidney function.

#### 3.4.8 Publication bias

The results of the publication bias assessment for other analyses are presented in Supplementary Figures S24–32. Funnel plots indicated no obvious evidence of publication bias.

## 4 Discussion

This review presents a comprehensive summary of the epidemiology, clinical outcomes, and treatment patterns of CMV infection among allo-HSCT recipients in China. Our findings indicate a substantial incidence of CMV infection in Chinese recipients of allo-HSCT (48.87%), which may vary according to the follow-up period, patient's age, transplantation type, and donor and recipient's CMV serologic status.

The incidence rates of CMV infection after allo-HSCT vary globally, ranging from 24.6 to 62.6% in North America, 28.9 to 63.9% in Europe, and 24.9 to 61.2% in other countries (Bergamasco et al., 2021; Cho et al., 2023). Our review found that the pooled incidence of CMV infection in Chinese recipients aligns with global trends and is at the higher end of values. This may be attributed to the presence of high risk factors in China, including the considerable prevalence of haplo-HSCT (60.1%) (Xu et al., 2021) and the high proportion of R+ individuals indicated by the high general incidence of CMV infection (>90%) in the Chinese population (Stem Cell Application Group, Chinese Society of Hematology, Chinese Medical Association, 2022). Consistent with the findings of the 2022 Chinese consensus (Stem Cell Application Group, Chinese Society of Hematology, Chinese Medical Association, 2022) and published evidence in Europe (Huntley et al., 2020; Solano et al., 2021) and North America (Huang et al., 2019; Webb et al., 2018) regarding risk factors of CMV infection after allo-HSCT, our review found that D–/R+ serostatus (57.64%), haplo-HSCT (69.08%) and unrelated-matched HSCT (70.82%) exhibited higher incidence rates than other serostatus conditions and transplant types. This underscores the necessity for tailored and culturally sensitive strategies to effectively mitigate risks associated with CMV. Notably, the high seroprevalence of CMV infection in China indicates that most patients have been exposed to the virus. Therefore, even with antiviral prophylaxis, reactivation of the virus may still occur, leading to breakthrough infections. Our results show that the rate of breakthrough infections is 7.29%. This highlights the need for regular monitoring of patients during prophylaxis, allowing for timely pre-emptive treatment upon detection of CMV reactivation to prevent progression to CMV disease or other complications.

In addition, the pooled incidence of CMV disease among recipients with CMV infection in China of 13.40% (95% CI 9.14–19.65%) is consistent with the estimates from other countries (2.9–15.7%) (Cho et al., 2023). In contrast to previous research findings, according to which CMV pneumonitis is the most frequent and common cause of death (Ljungman et al., 2019), our results indicate that the incidence of CMV pneumonitis (5.96%) was lower than that of CMV cystitis (13.48%) and CMV enteritis (6.62%). This unexpected result may stem from methodological variations across the included studies, which may indicate differences in study quality, diagnostic criteria and immunosuppressive treatment regimens across the included studies.

Moreover, our review found that 32.03% of recipients with CMV infection experienced refractory CMV, suggesting that some patients have inadequate responses to conventional treatment (Chemaly et al., 2019). Given that refractory CMV infection is recognized as an independent risk factor for CMV diseases and non-relapse mortality (Liu et al., 2015), concurrently increasing the risk of developing GVHD (Nho et al., 2023), the relatively high incidence may emphasize the need for heightened clinical attention after allo-HSCT. The high refractory CMV infections may be influenced by a variety of factors that can be broadly categorized into patient-related factors (e.g., immune status, serological status, transplant type), virus-related factors (e.g., High viral load), and drug-related factors (e.g., antiviral resistance, drug exposure). These factors interact in complex ways, making the management of refractory CMV infections particularly challenging. Our analysis of the included studies indicates that it is challenging to distinguish CMV infection rates due to the heterogeneity of the studies and the lack of detailed information on the specific drugs and regimens used. Thus, further large-scale prospective studies are warranted. Our review also obtained a rate of 15.42% for CMV recurrence after allo-HSCT among recipients with CMV infection. The refractory and/or recurrent CMV infection may be attributed to factors including mismatched donor transplants, T-cell depletion and CMV-seropositive recipients (Gagelmann et al., 2018; Liu et al., 2015; Almyroudis et al., 2007). This information indicates to clinicians the ongoing risk of refractory and/or recurrence CMV for recipients with CMV infection after allo-HSCT. Furthermore, the absence of resistance data suggests a potential lack of emphasis on resistance in clinical practice. Limited access to resistance detection methods may lead to an underestimation of the resistance incidence. It is crucial to accumulate more resistance data to enhance understanding of CMV resistance and to guide individualized treatment strategies.

The findings of this review indicate that ganciclovir, valganciclovir and foscarnet currently remain the most commonly used drugs in both prophylaxis and pre-emptive therapy in China. Despite their widespread use, these medications are associated with treatment-limiting toxicity. Our results reveal that a notable proportion of patients treated with ganciclovir and foscarnet experience myelosuppression (34.90–41.20%) and kidney function impairment (11.4%). The treatment related myelosuppression can directly impact the generation of cytotoxic T lymphocytes (CTL), leading to a delayed immune reconstitution associated with CTL (Martín-Gandul et al., 2014). Additionally, neutropenia may increase the risk of opportunistic infections and bleeding (Qi et al., 2022; Scott et al., 2008). Furthermore, foscarnet-induced nephrotoxicity can impact drug absorption and metabolism, potentially leading to conditions such as acute kidney injury and even uraemia in severe cases (Inose et al., 2022). The findings on treatment patterns highlight the unmet medical need in China for developing more effective and well-tolerated alternatives to fill the existing treatment gaps of CMV infection. With the availability of the novel drug letermovir, the treatment pattern in China is shifting from pre-emptive therapy toward prophylaxis therapy, although data on its efficacy and safety in the Chinese population remains limited (Stem Cell Application Group, Chinese Society of Hematology, Chinese Medical Association, 2022). A recent study published in 2023 supports the potential benefits of letermovir in reducing the incidence of CMV infection after haplo-HSCT without increasing the risks of aGVHD, non-relapse mortality and myelosuppression (Ma et al., 2023a). However, it is noteworthy that 17.6% of patients in the 2023 study still experienced CMV reactivation after discontinuation of letermovir, which may due to letermovir postponing CMV-specific immune reconstitution (Gabanti et al., 2022). It is worth mentioning that the recent approval of maribavir in China may offer a significant advancement in the treatment of post-transplant refractory CMV infections or diseases (PR Newswire, 2023). In the phase 3 RCT, maribavir exhibited greater efficacy than valganciclovir/ganciclovir, foscarnet, or cidofovir in CMV viremia resolution (55.7 vs. 23.9%) (Avery et al., 2022). This was coupled with a lower incidence of treatment-related acute kidney injury compared to foscarnet (1.7 vs. 19.1%) and reduced treatment-related neutropenia compared to valganciclovir/ganciclovir (1.7% vs. 25%) (Avery et al., 2022). The favorable efficacy and reduced treatment-related adverse events have the potential to address the current unmet treatment needs in this context (2023). Additionally, the development of CMV vaccines is progressing, with an mRNA-based vaccine demonstrating strong immune responses in laboratory studies (Fierro et al., 2024). A second-generation CMV vaccine candidate, T10-F10 (Yll-Pico et al., 2024), developed using a synthetic poxvirus platform, has shown high stability and immunogenicity in preclinical studies. CMV-specific T-cell therapies are also being explored as alternative strategies (Tang et al., 2024), with recent studies highlighting their potential in managing CMV infections. Future research should focus on optimizing these existing strategies and exploring new avenues.

Our study has several limitations. Despite efforts to include studies without restrictions on publication status, publication bias may still exist, as the included studies were identified using specific keywords in bibliographical databases. The exclusion of non-English and non-Chinese articles may lead to bias. This practice, known as language bias, can limit the generalizability of the findings and potentially skew the results by omitting key data from studies published in other languages. Moreover, the substantial heterogeneity observed across outcomes underscores the need for more homogeneous study designs and reporting standards in future research. Investigating the sources of this heterogeneity is essential to better understand the factors driving variations in CMV infection rates after allo-HSCT. These rates vary widely due to patient characteristics (e.g., CMV serostatus, age, underlying diseases), transplant type, immunosuppressive regimens, monitoring methods, geographic and demographic differences, post-transplant complications (e.g., GVHD), and variations in healthcare practices. The lack of a unified threshold for pre-emptive CMV treatment further contributes to this variability. Addressing these issues is critical to improving clinical management and advancing research in this field. In addition, we conducted a proportional meta-analysis, which restricted our ability to investigate the association between subgroup factors and the incidence of CMV infection. Future research could explore these potential confounders more comprehensively to provide a more detailed understanding of their impact on outcomes. Proportional meta-analysis synthesizes proportions from multiple studies into a pooled estimate, focusing on binary outcomes like CMV infection rates (Barker et al., 2021). However, this method limits the exploration of how subgroup factors, such as patient demographics, transplant characteristics, or treatment protocols, influence CMV infection rates. Therefore, further studies are warranted to investigate whether these subgroup factors contribute to the risk of CMV infection in recipients following allo-HSCT.

This review consolidates evidence on the epidemiology, clinical outcomes, and treatment patterns in recipients following allo-HSCT in China. Our results highlight a notable incidence of CMV infection in this population, with a portion of patients developing refractory CMV infection. Current anti-CMV therapies are limited and often associated with treatment-limiting toxicities, emphasizing the need for more effective and better-tolerated treatment options. Furthermore, the development of an effective CMV vaccine could further enhance the prevention and management of CMV infections in this high-risk population.

## Data Availability

The raw data supporting the conclusions of this article will be made available by the authors, without undue reservation.
