# Supplementary material for: Epidemiology, clinical outcomes, and treatment patterns of cytomegalovirus infection after allogeneic hematopoietic stem cell transplantation in China: a scoping review and meta-analysis
Source: Front Microbiol. 2025 Apr 3;16:1518275. doi: 10.3389/fmicb.2025.1518275 (PMC12003426; doi:10.3389/fmicb.2025.1518275)
Supplement: Supplementary file 1 [file Data_Sheet_1.pdf]

Supplementary files

Contents

Supplementary 1 Search strategy 2
Supplementary 2 Quality assessment results of included studies 6
See EXCEL supplementary 2 table S1-table S4 6
Supplementary 3 Forest plots for meta-analyses results 6
Incidence of breakthrough CMV infection 6
Incidence of CMV infection based on various types of transplantation 6
Incidence of CMV infection based on prior-transplantation CMV serologic status of donor and recipients 8
Incidence of cumulative CMV infection within various follow-up periods 9
Incidence of CMV infection based on patient’s age 11
Incidence of refractory and recurrent CMV infection 11
Incidence of CMV diseases 12
Mortality rate 14
Incidence of comorbidities 15
Supplementary 4 Funnel plots assessing publication bias on the results with 10 or more included studies 16

Supplementary 1 Search strategy
PubMed

#1. "Cytomegalovirus"[Mesh] OR "Cytomegalovirus Infections"[Mesh] OR Cytomegalovirus*[tw] OR cytomegalus virus*[tw] OR cytomegalusvirus*[tw] OR "Salivary Gland Virus*"[tw] OR "Human Herpesvirus 5"[tw] OR "HHV 5"[tw] OR Cytomegalic[tw] OR CMV[tw] OR HCMV[tw] OR Cytomegaly[tw] OR cytomegalia[tw] OR cytomegalo[tw] OR cytomegalus[tw] OR cytomegalusvirus*[tw] OR "human herpes virus 5"[tw] OR cytomegaloinfection*[tw] OR cytomegaloviral[tw]

#2. "Stem Cell Transplantation"[Mesh] OR "Bone Marrow Transplantation"[Mesh] OR "Bone Marrow Grafting"[tw] OR "Bone Marrow Cell Transplant*"[tw] OR "Bone Marrow Transplant*"[tw] OR "stem cell Transplant*"[tw] OR "hematopoietic cell Transplant*"[tw] OR "stem cell Grafting"[tw] OR "Haploidentical Transplant*"[tw] OR "unrelated donor Transplant*"[tw] OR "Cord Blood Stem Cell Transplantation"[Mesh] OR "Cord Blood Transplant*"[tw] OR "Umbilical cord blood Transplant*"[tw] OR "Umbilical blood Transplant*"[tw] OR "Semicongruent Transplant*"[tw] OR Halfmatched[tw] OR "Half matched"[tw] OR "allo-HSCT"[tw] OR Peripheral blood Transplant*[tw] OR "matched sibling donor"[tw] OR "matched unrelated donor"[tw] OR "haplo transplantation*"[tw] OR "haploidentical transplantation*"[tw] OR "matched related donor"[tw]

#3. #1 AND #2

#4. "China"[Mesh] OR China OR Chinese OR Taiwan OR Taiwanese OR "Hong kong" OR Hongkong OR Macau OR Macao OR Beijing OR Shanghai OR Tianjin OR Chongqing OR "Inner Mongolia" OR Tibet OR Guangxi OR Sinkiang OR Ningxia OR Xinjiang OR Hebei OR Shanxi OR Liaoning OR Jilin OR Heilongjiang OR Jiangsu OR Zhejiang OR Anhui OR Fujian OR Jiangxi OR Shandong OR Henan OR Hubei OR Hunan OR Guangdong OR Hainan OR Sichuan OR Guizhou OR Yunnan OR Shaanxi OR Gansu OR Qinghai

#5. "Review Literature as Topic"[Mesh] OR "Review" [Publication Type] OR Review[ti] OR "Case Reports as Topic"[Mesh] OR "Case Reports" [Publication Type] OR "case report"[ti] OR "a case"[ti] OR ("Animals"[Mesh] NOT ("Humans"[Mesh] AND "Animals"[Mesh]))

#6. "Meta-Analysis"[pt] OR "Meta-Analysis as Topic"[Mesh] OR "Systematic Review" [pt] OR "Systematic Reviews as Topic"[Mesh] OR "systematic"[Filter] OR "Systematic Review*"[tiab] OR "Meta-Analysis"[tiab] OR Metaanalys*[tiab]

#7. (#3 AND #4) NOT (#5 NOT #6)

EMBASE

#1. 'Cytomegalovirus'/exp OR 'cytomegalovirus infection'/exp OR (Cytomegalovirus* OR "cytomegalus virus*" OR cytomegalusvirus* OR "Salivary Gland Virus*" OR "Human Herpesvirus 5" OR "HHV 5" OR Cytomegalic OR CMV OR HCMV OR Cytomegaly OR cytomegalia OR cytomegalo OR cytomegalus OR cytomegalusvirus* OR "human herpes virus 5" OR cytomegaloinfection* OR cytomegaloviral):ab,ti,kw

#2. 'stem cell transplantation'/exp OR 'bone marrow transplantation'/exp OR ((("Stem Cell*" OR "Bone Marrow" OR "hematopoietic cell*" OR Haploidentical OR "unrelated donor*" OR "Cord Blood" OR "Umbilical blood" OR Semicongruent OR Halfmatched OR "Half matched" OR "allo-HSCT" OR "Peripheral blood" OR "matched sibling donor*" OR haplo OR haploidentical OR "related donor*") NEAR/3 (Grafting* OR Transplant*))):ab,ti,kw

#3. #1 AND #2

#4. 'China'/exp OR (China OR Chinese OR Taiwan OR "Hong kong" OR Hongkong OR Macau OR Macao OR Beijing OR Shanghai OR Tianjin OR Chongqing OR "Inner Mongolia" OR Tibet OR Guangxi OR Sinkiang OR Ningxia OR Xinjiang OR Hebei OR Shanxi OR Liaoning OR Jilin OR Heilongjiang OR Jiangsu OR Zhejiang OR Anhui OR Fujian OR Jiangxi OR Shandong OR Henan OR Hubei OR Hunan OR Guangdong OR Hainan OR Sichuan OR Guizhou OR Yunnan OR Shaanxi OR Gansu OR Qinghai):ti,ab,ad,ff

#5. 'review'/exp OR 'Case Report'/exp OR 'conference abstract'/it OR 'editorial'/it OR 'letter'/it OR 'review'/it OR (('nonhuman'/exp OR 'animal'/exp) NOT 'human'/exp) OR (Review OR "case report" OR "a case"):ti

#6. 'meta analysis'/exp OR 'meta analysis (topic)'/exp OR 'systematic review'/exp OR 'systematic review (topic)'/exp OR (Systemat* NEAR/3 Review* OR 'Meta Analysis*' OR Metaanalys*):ab,ti,kw

#7. (#3 AND #4) NOT (#5 NOT #6)

Web of Science

#1. TS=(Cytomegalovirus* OR "cytomegalus virus*" OR cytomegalusvirus* OR "Salivary Gland Virus*" OR "Human Herpesvirus 5" OR "HHV 5" OR Cytomegalic OR CMV OR HCMV OR Cytomegaly OR cytomegalia OR cytomegalo OR cytomegalus OR cytomegalusvirus* OR "human herpes virus 5" OR cytomegaloinfection* OR cytomegaloviral)

#2. TS=((("Stem Cell*" OR "Bone Marrow" OR "hematopoietic cell*" OR Haploidentical OR "unrelated donor*" OR "Cord Blood" OR "Umbilical blood" OR Semicongruent OR Halfmatched OR "Half matched" OR "allo-HSCT" OR "Peripheral blood" OR "matched sibling donor*" OR haplo OR haploidentical OR "related donor*") NEAR/3 (Grafting* OR Transplant*)))

#3. #1 AND #2 6708

#4. CU=China OR TS=(China OR Chinese OR Taiwan OR "Hong kong" OR Hongkong OR Macau OR Macao OR Beijing OR Shanghai OR Tianjin OR Chongqing OR "Inner Mongolia" OR Tibet OR Guangxi OR Sinkiang OR Ningxia OR Xinjiang OR Hebei OR Shanxi OR Liaoning OR Jilin OR Heilongjiang OR Jiangsu OR Zhejiang OR Anhui OR Fujian OR Jiangxi OR Shandong OR Henan OR Hubei OR Hunan OR Guangdong OR Hainan OR Sichuan OR Guizhou OR Yunnan OR Shaanxi OR Gansu OR Qinghai) OR AD=(China OR Chinese OR Taiwan OR "Hong kong" OR Hongkong OR Macau OR Macao OR Beijing OR Shanghai OR Tianjin OR Chongqing OR "Inner Mongolia" OR Tibet OR Guangxi OR Sinkiang OR Ningxia OR Xinjiang OR Hebei OR Shanxi OR Liaoning OR Jilin OR Heilongjiang OR Jiangsu OR Zhejiang OR Anhui OR Fujian OR Jiangxi OR Shandong OR Henan OR Hubei OR Hunan OR Guangdong OR Hainan OR Sichuan OR Guizhou OR Yunnan OR Shaanxi OR Gansu OR Qinghai)

#5. TI=(Review OR "case report" OR "a case" OR animal* OR rat OR rats OR mouse OR mice OR rabbit* OR dog* OR cat* OR pig* OR swine* OR monkey* OR sheep* OR goat*)

#6. TS=("Meta-Analysis" OR "Meta Analysis" OR "Systematic Review" OR "Systematic Review*" OR "Meta-Analysis" OR "Meta-Analy*" OR "Metaanaly*" OR ("systematic*" NEAR/4 "Review*"))

#7. (#3 AND #4) NOT (#5 NOT #6)

Cochrane Library

#1	MeSH descriptor: [Cytomegalovirus] explode all trees

#2	MeSH descriptor: [Cytomegalovirus Infections] explode all trees

#3	(Cytomegalovirus* OR "cytomegalus virus*" OR cytomegalusvirus* OR "Salivary Gland Virus*" OR "Human Herpesvirus 5" OR "HHV 5" OR Cytomegalic OR CMV OR HCMV OR Cytomegaly OR cytomegalia OR cytomegalo OR cytomegalus OR cytomegalusvirus* OR "human herpes virus 5" OR cytomegaloinfection* OR cytomegaloviral):ti,ab,kw

#4	#1 OR #2 OR #3

#5	MeSH descriptor: [Stem Cell Transplantation] explode all trees	2872

#6	MeSH descriptor: [Bone Marrow Transplantation] explode all trees

#7	((("Stem Cell*" OR "Bone Marrow" OR "hematopoietic cell*" OR Haploidentical OR "unrelated donor*" OR "Cord Blood" OR "Umbilical blood" OR Semicongruent OR Halfmatched OR "Half matched" OR "allo-HSCT" OR "Peripheral blood" OR "matched sibling donor*" OR haplo OR haploidentical OR "related donor*") NEAR/3 (Grafting* OR Transplant*))):ti,ab,kw

#8	#5 OR #6 OR #7

#9	MeSH descriptor: [China] explode all trees

#10	China OR Chinese OR Taiwan OR Taiwanese OR "Hong kong" OR Hongkong OR Macau OR Macao OR Beijing OR Shanghai OR Tianjin OR Chongqing OR "Inner Mongolia" OR Tibet OR Guangxi OR Sinkiang OR Ningxia OR Xinjiang OR Hebei OR Shanxi OR Liaoning OR Jilin OR Heilongjiang OR Jiangsu OR Zhejiang OR Anhui OR Fujian OR Jiangxi OR Shandong OR Henan OR Hubei OR Hunan OR Guangdong OR Hainan OR Sichuan OR Guizhou OR Yunnan OR Shaanxi OR Gansu OR Qinghai

#11	#9 OR #10	124780

#12	#4 AND #8 AND #11

CNKI（期刊、学位、会议，中英文扩展：是，中文）

((SU%=巨细胞病毒+人巨细胞病毒+巨细胞包涵体病+CMV+HCMV OR TKA=巨细胞病毒+人巨细胞病毒+巨细胞包涵体病+CMV+HCMV) AND (SU%=移植*(干细胞+骨髓+血液+半相合+脐带血+脐血+外周血) OR TKA=移植*(干细胞+骨髓+血液+半相合+脐带血+脐血+外周血))) NOT TKA=鼠+大鼠+小鼠+雌鼠+猴+猪+羊+兔

万方

(主题:("巨细胞病毒" OR "巨细胞包涵体病" OR "CMV" OR "HCMV") and 主题:("移植") and 主题:(干细胞 OR 骨髓 OR 血液 OR 半相合 OR 脐带血 OR 脐血 OR 外周血)) not 题名:(鼠 OR 猴 OR 猪 OR 羊 OR 兔)	717

(主题:("巨细胞病毒" OR "巨细胞包涵体病" OR "CMV" OR "HCMV") and 主题:("移植") and 主题:("干细胞" OR "骨髓" OR "血液" OR "半相合" OR "脐带血" OR "脐血" OR "外周血")) not 题名:(鼠 OR 猴 OR 猪 OR 羊 OR 兔)

CBM

(("巨细胞病毒"[常用字段:智能] OR "人巨细胞病毒"[常用字段:智能] OR "巨细胞包涵体病"[常用字段:智能] OR "CMV"[常用字段:智能] OR "HCMV"[常用字段:智能]) AND "移植"[常用字段:智能] AND ("干细胞"[常用字段:智能] OR "骨髓"[常用字段:智能] OR "血液"[常用字段:智能] OR "半相合"[常用字段:智能] OR "脐带血"[常用字段:智能] OR "脐血"[常用字段:智能] OR "外周血"[常用字段:智能])) NOT ("鼠"[中文标题:智能] OR "大鼠"[中文标题:智能] OR "小鼠"[中文标题:智能] OR "雌鼠"[中文标题:智能] OR "猴"[中文标题:智能] OR "猪"[中文标题:智能] OR "羊"[中文标题:智能] OR "兔"[中文标题:智能])

Supplementary 2 Quality assessment results of included studies

See EXCEL supplementary 2 table S1-table S4

Supplementary 3 Forest plots for meta-analyses results

Incidence of breakthrough CMV infection

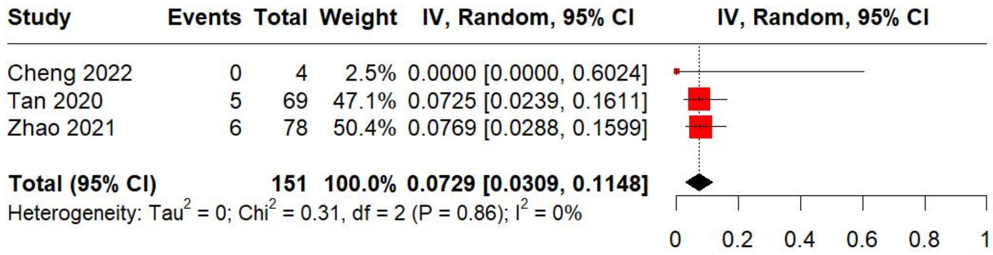

Figure S1 Forest plot of incidence of breakthrough cytomegalovirus (CMV) infection after allogeneic hematopoietic stem cell transplantation

Incidence of CMV infection based on various types of transplantation

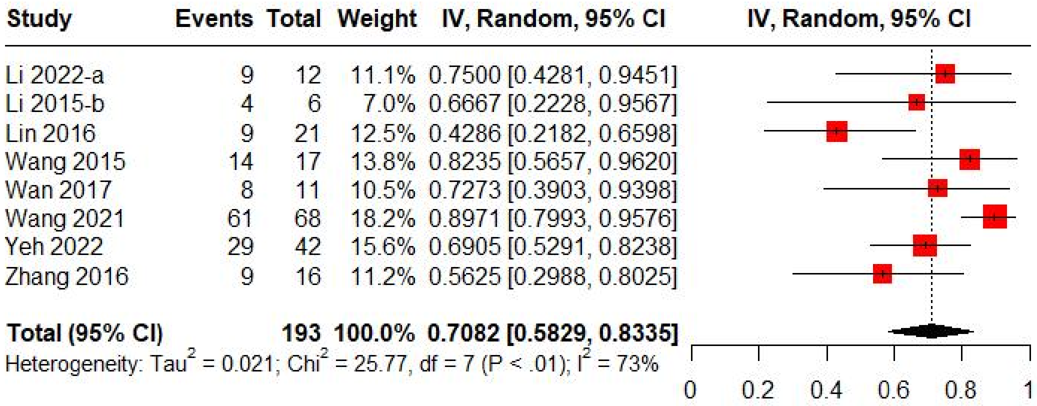

Figure S2 Forest plot of incidence of CMV infection after unrelated matched allogeneic hematopoietic stem cell transplantation

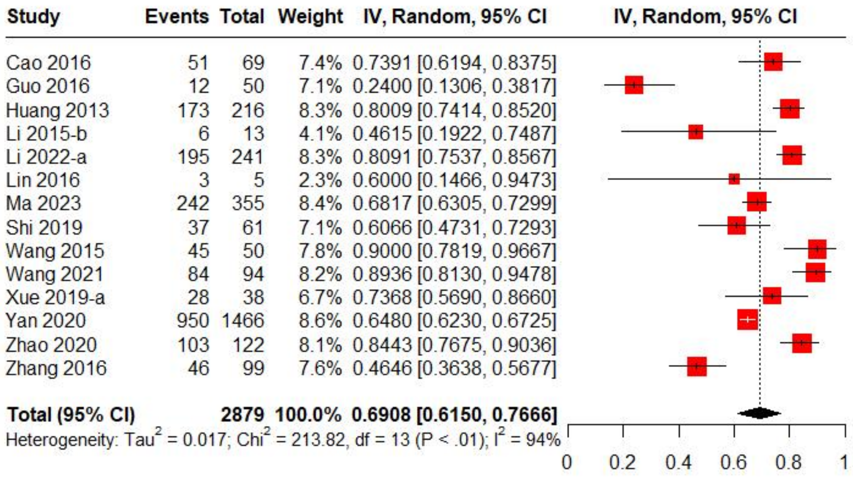

Figure S3 Forest plot of incidence of CMV infection after haploidentical allogeneic hematopoietic stem cell transplantation

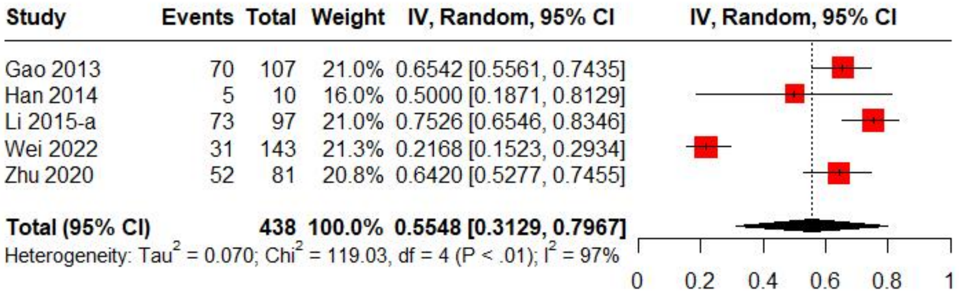

Figure S4 Forest plot of incidence of CMV infection after cord blood allogeneic hematopoietic stem cell transplantation

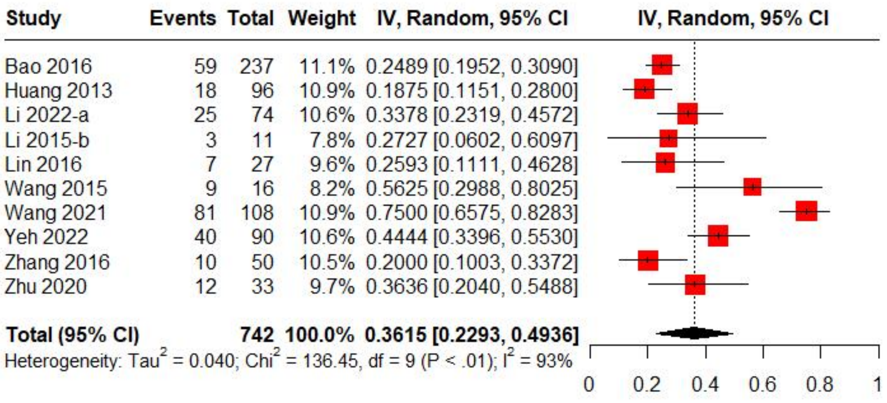

Figure S5 Forest plot of incidence of CMV infection after human leukocyte antigen-matched allogeneic hematopoietic stem cell transplantation

Incidence of CMV infection based on prior-transplantation CMV serologic status of donor and recipients

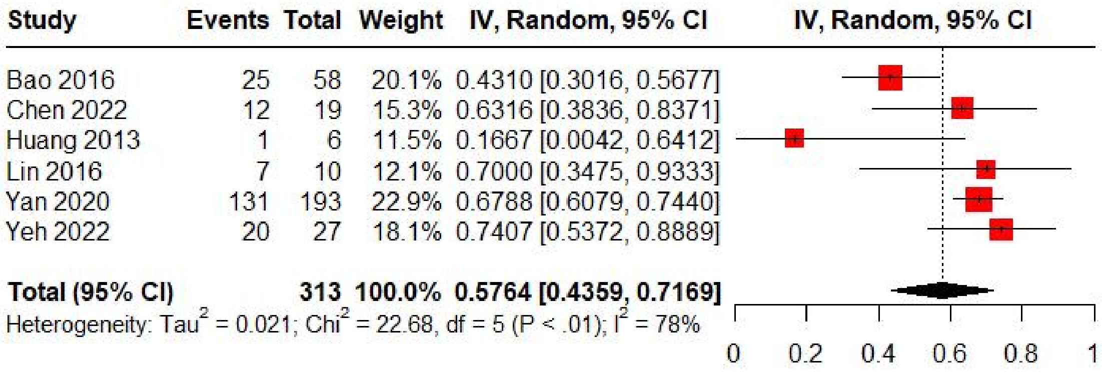

Figure S6 Forest plot of incidence of CMV infection after allogeneic hematopoietic stem cell transplantation based on the prior-transplantation serologic status of donor (negative) and recipient (positive)

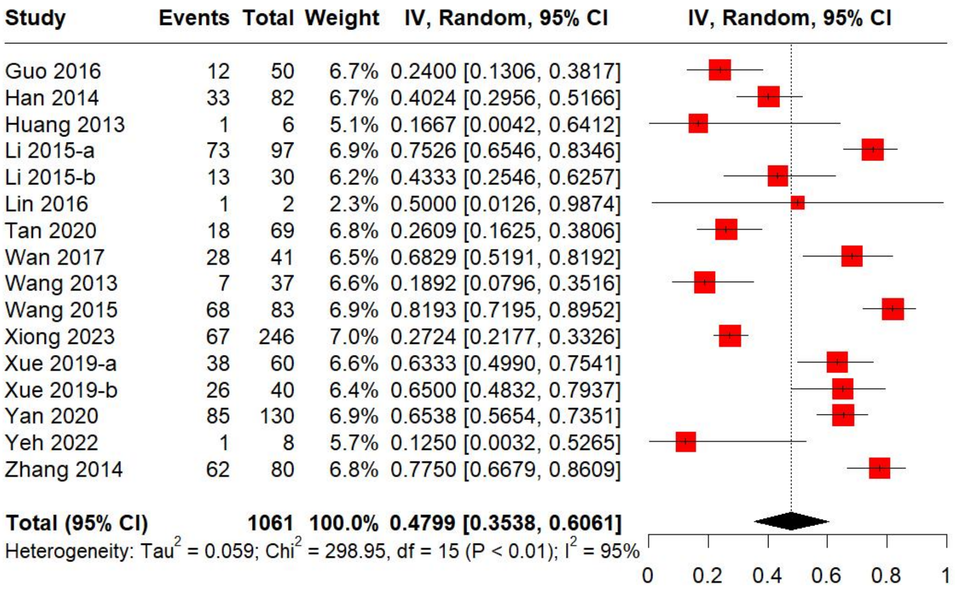

Figure S7 Forest plot of incidence of CMV infection after allogeneic hematopoietic stem cell transplantation based on the prior-transplantation serologic status of donor (negative) and recipient (negative)

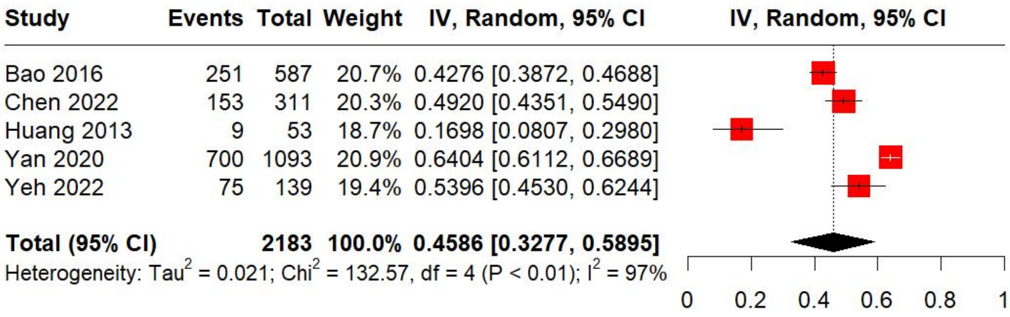

Figure S8 Forest plot of incidence of CMV infection after allogeneic hematopoietic stem cell transplantation based on the prior-transplantation serologic status of donor (positive) and recipient (positive)

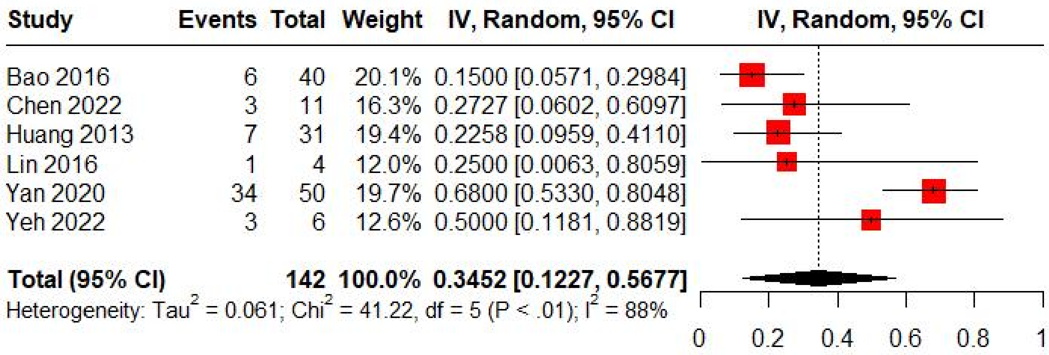

Figure S9 Forest plot of incidence of CMV infection after allogeneic hematopoietic stem cell transplantation based on the prior-transplantation serologic status of donor (positive) and recipient (negative)

Incidence of cumulative CMV infection within various follow-up periods

Study	Events	Total	Weight	IV, Random, 95% CI
Bi 2015	8	65	5.3%	0.1231 [0.0547, 0.2282]
Cao 2016-b	49	69	5.2%	0.7101 [0.5884, 0.8131]
Chen 2022	173	359	5.3%	0.4819 [0.4291, 0.5349]
Ding 2021	3	160	5.3%	0.0187 [0.0039, 0.0538]
Gao 2013	70	107	5.3%	0.6542 [0.5561, 0.7435]
Huang 2022	67	257	5.3%	0.2607 [0.2081, 0.3189]
Li 2013-a	113	141	5.3%	0.8014 [0.7259, 0.8638]
Li 2021	35	84	5.2%	0.4167 [0.3100, 0.5294]
Shi 2019	37	61	5.2%	0.6066 [0.4731, 0.7293]
Tan 2020	18	69	5.2%	0.2609 [0.1625, 0.3806]
Wan 2017	27	41	5.1%	0.6585 [0.4941, 0.7992]
Wang 2015	68	83	5.3%	0.8193 [0.7195, 0.8952]
Wang 2021	229	270	5.3%	0.8481 [0.7997, 0.8888]
Wei 2022	31	143	5.3%	0.2168 [0.1523, 0.2934]
Wu 2019	21	53	5.2%	0.3962 [0.2645, 0.5400]
Yeh 2022	99	180	5.3%	0.5500 [0.4742, 0.6241]
Zhang 2016	61	165	5.3%	0.3697 [0.2960, 0.4482]
Zhao 2021	18	78	5.3%	0.2308 [0.1429, 0.3400]
Zhu 2020	162	269	5.3%	0.6022 [0.5410, 0.6612]
**Total (95% CI)**		**2654**	**100.0%**	**0.4746 [0.3190, 0.6302]**

Heterogeneity: $Tau^2 = 0.118$; $Chi^2 = 2078.17$, df = 18 (P = 0); $I^2 = 99\%$

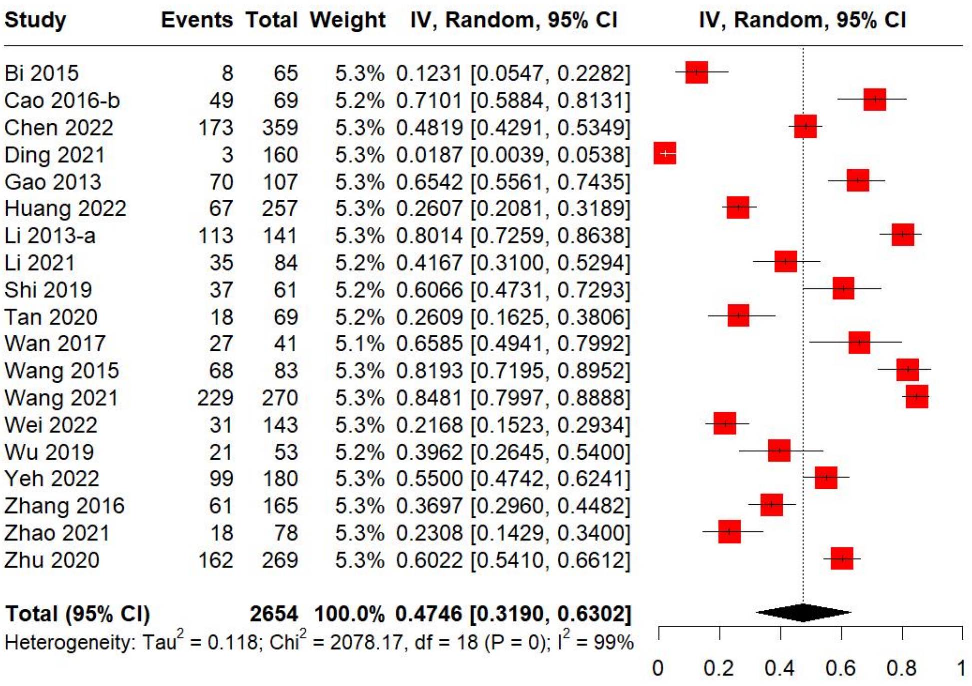

Figure S10 Forest plot of incidence of cumulative CMV infection within 100 days after allogeneic hematopoietic stem cell transplantation

Study	Events	Total	Weight	IV, Random, 95% CI
Li 2013-a	115	141	27.3%	0.8156 [0.7416, 0.8759]
Li 2015-b	13	30	19.5%	0.4333 [0.2546, 0.6257]
Zhang 2014	62	80	25.7%	0.7750 [0.6679, 0.8609]
Zhu 2020	165	269	27.6%	0.6134 [0.5523, 0.6719]
**Total (95% CI)**		**520**	**100.0%**	**0.6750 [0.5385, 0.8114]**

Heterogeneity: $Tau^2 = 0.017$; $Chi^2 = 32.25$, df = 3 (P < .01); $I^2 = 91\%$

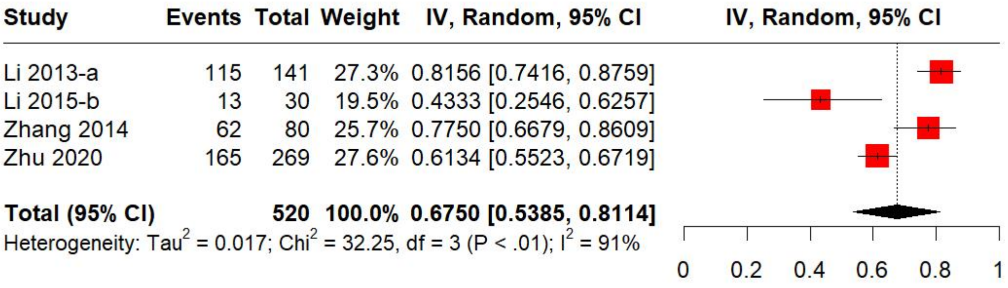

Figure S11 Forest plot of incidence of cumulative CMV infection within 200 days after allogeneic hematopoietic stem cell transplantation

Incidence of CMV infection based on patient's age

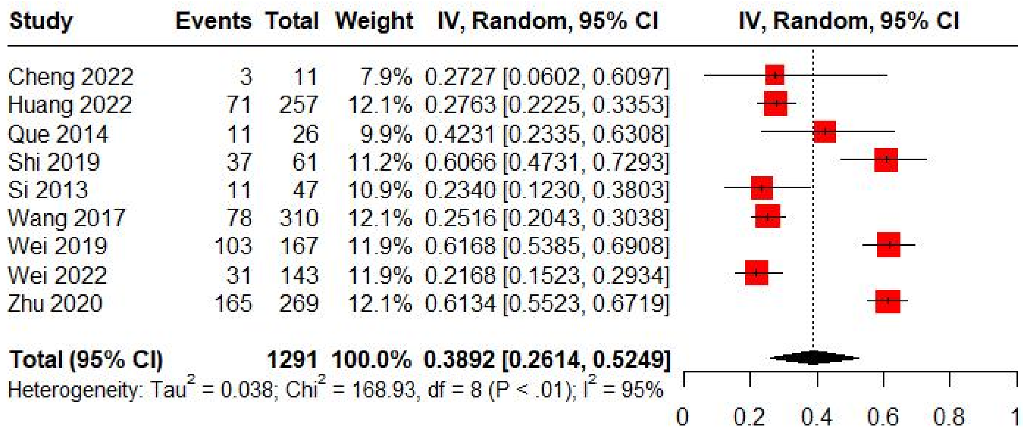

Figure S12 Forest plot of incidence of CMV infection in child recipients after allogeneic hematopoietic stem cell transplantation

Incidence of refractory and recurrent CMV infection

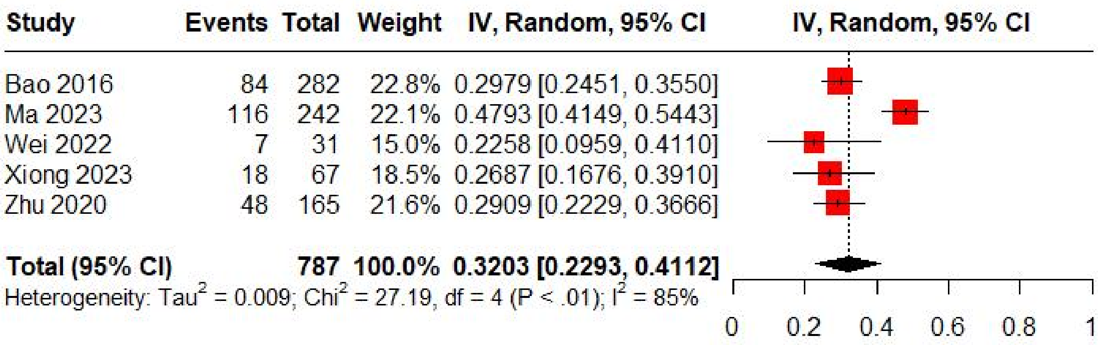

Figure S13 Forest plot of incidence of refractory CMV infection after allogeneic hematopoietic stem cell transplantation among recipients with CMV infection

Study	Events	Total	Weight	IV, Random, 95% CI
Han 2014	1	33	5.6%	0.0303 [0.0008, 0.1576]
Li 2013-a	10	115	9.3%	0.0870 [0.0425, 0.1541]
Li 2015-b	1	13	5.7%	0.0769 [0.0019, 0.3603]
Tan 2020	3	18	8.1%	0.1667 [0.0358, 0.4142]
Wang 2015	63	68	10.0%	0.9265 [0.8367, 0.9757]
Wang 2017	16	78	9.6%	0.2051 [0.1220, 0.3116]
Wei 2022	6	31	9.0%	0.1935 [0.0745, 0.3747]
Xiong 2023	3	67	7.9%	0.0448 [0.0093, 0.1253]
Xue 2019-b	2	26	7.3%	0.0769 [0.0095, 0.2513]
Zhang 2016	17	65	9.6%	0.2615 [0.1603, 0.3854]
Zhao 2021	3	18	8.1%	0.1667 [0.0358, 0.4142]
Zhu 2020	39	165	9.8%	0.2364 [0.1738, 0.3086]
**Total (95% CI)**		**697**	**100.0%**	**0.1542 [0.0776, 0.3063]**

Heterogeneity: $Tau^2 = 1.229$; $Chi^2 = 298.88$, df = 11 ($P < .01$); $I^2 = 96\%$

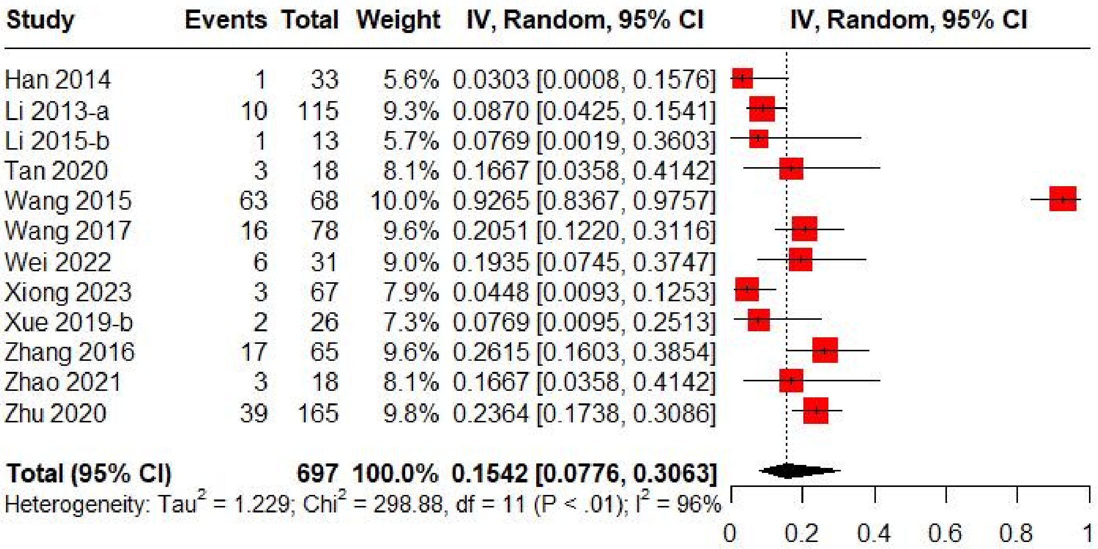

Figure S14 Forest plot of incidence of recurrent CMV infection after allogeneic hematopoietic stem cell transplantation among recipients with CMV infection

Incidence of CMV diseases

Study	Events	Total	Weight	IV, Random, 95% CI
He 2014	17	108	5.5%	0.1574 [0.0945, 0.2400]
Huang 2013	19	216	5.5%	0.0880 [0.0538, 0.1340]
Jin 2014	4	115	3.7%	0.0348 [0.0096, 0.0867]
Kong 2015	31	488	5.8%	0.0635 [0.0436, 0.0890]
Lin 2018	1	31	1.7%	0.0323 [0.0008, 0.1670]
Meng 2020	113	3862	6.1%	0.0293 [0.0242, 0.0351]
Que 2014	2	26	2.7%	0.0769 [0.0095, 0.2513]
Si 2013	2	47	2.7%	0.0426 [0.0052, 0.1454]
Tan 2020	4	69	3.8%	0.0580 [0.0160, 0.1418]
Tong 2013	4	176	3.7%	0.0227 [0.0062, 0.0572]
Wang 2013	4	37	3.9%	0.1081 [0.0303, 0.2542]
Wang 2015	2	83	2.7%	0.0241 [0.0029, 0.0843]
Wang 2017	3	310	3.3%	0.0097 [0.0020, 0.0280]
Wang 2021	18	270	5.5%	0.0667 [0.0400, 0.1033]
Wu 2017	6	96	4.4%	0.0625 [0.0233, 0.1311]
Xue 2019-a	1	60	1.7%	0.0167 [0.0004, 0.0894]
Xue 2019-b	1	40	1.7%	0.0250 [0.0006, 0.1316]
Yeh 2022	9	180	4.8%	0.0500 [0.0231, 0.0928]
Yin 2021	30	361	5.8%	0.0831 [0.0568, 0.1165]
Zhang 2014	9	80	4.9%	0.1125 [0.0528, 0.2028]
Zhang 2021	8	73	4.8%	0.1096 [0.0485, 0.2046]
Zhao 2021	8	39	4.9%	0.2051 [0.0930, 0.3646]
Zhu 2020	9	269	4.8%	0.0335 [0.0154, 0.0626]
Zou 2016	33	398	5.8%	0.0829 [0.0578, 0.1145]
**Total (95% CI)**		**7434**	**100.0%**	**0.0596 [0.0444, 0.0800]**

Heterogeneity: $Tau^2 = 0.361$; $Chi^2 = 135.07$, df = 23 ($P < .01$); $I^2 = 83\%$

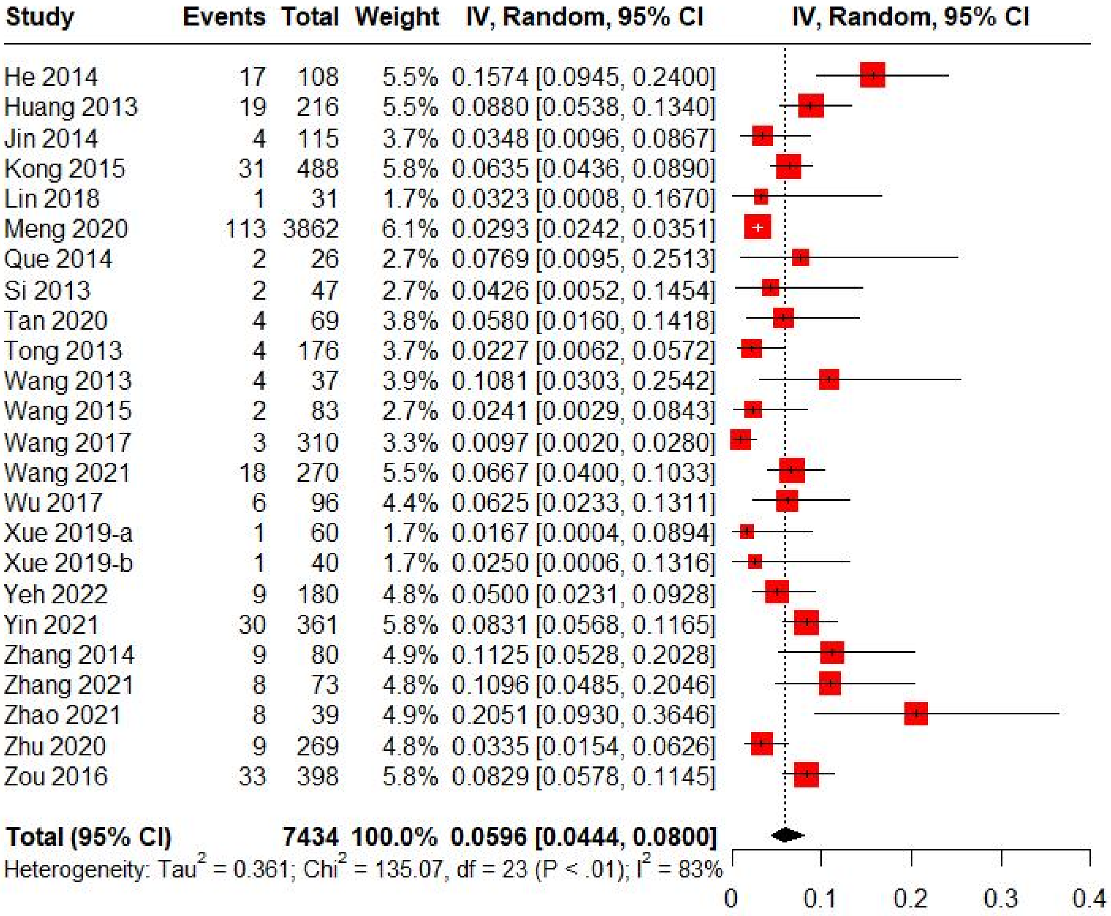

Figure S15 Forest plot of incidence of CMV pneumonitis after allogeneic hematopoietic stem cell transplantation among recipients with CMV infection

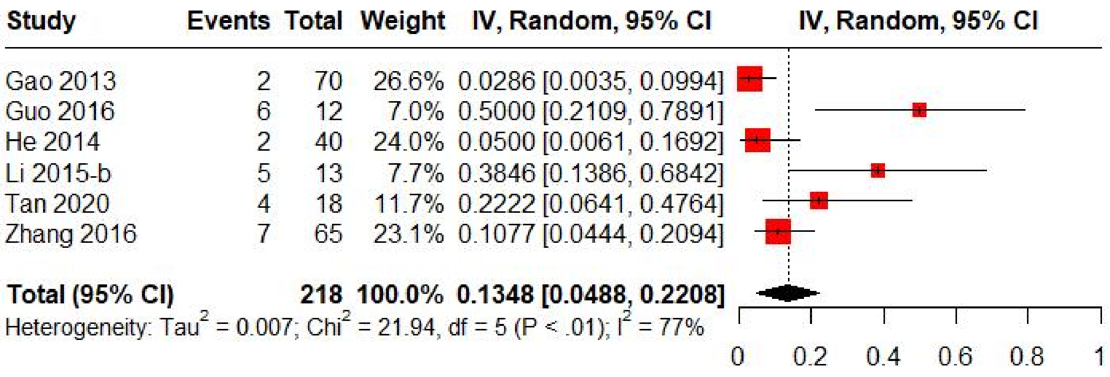

Figure S16 Forest plot of incidence of CMV cystitis after allogeneic hematopoietic stem cell transplantation among recipients with CMV infection

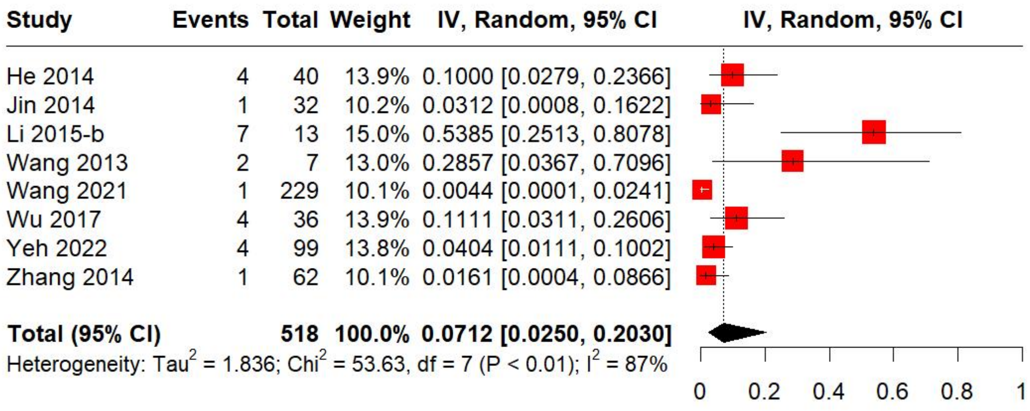

Figure S17 Forest plot of incidence of CMV enteritis after allogeneic hematopoietic stem cell transplantation among recipients with CMV infection

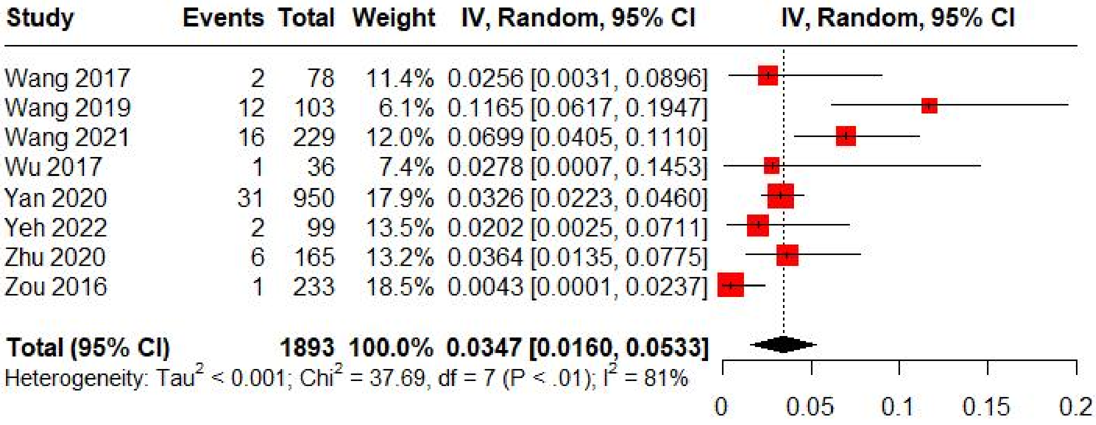

Figure S18 Forest plot of incidence of CMV retinitis after allogeneic hematopoietic stem cell transplantation among recipients with CMV infection

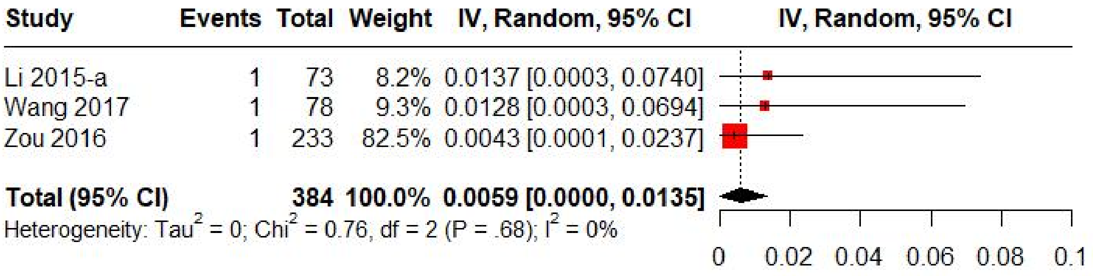

Figure S19 Forest plot of incidence of CMV encephalitis after allogeneic hematopoietic stem cell transplantation among recipients with CMV infection

Mortality rate

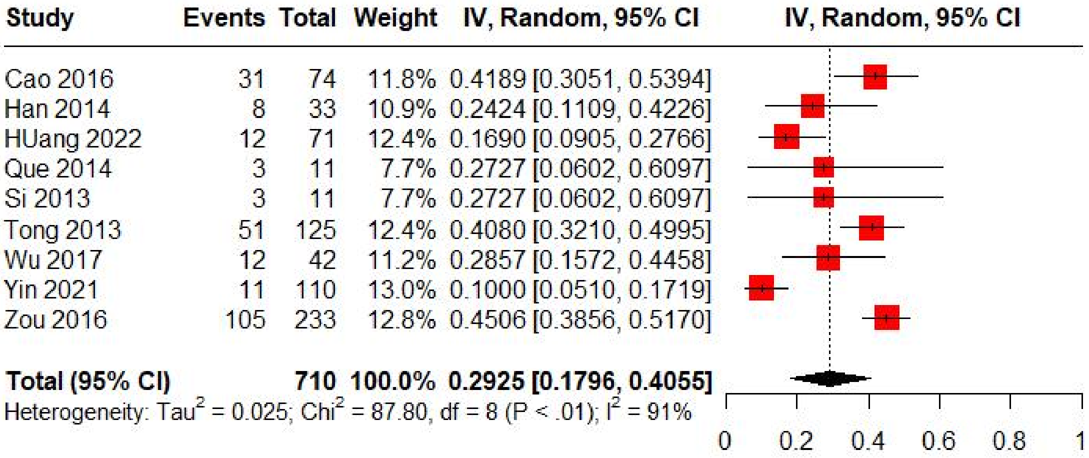

Figure S20 Forest plot of all-cause mortality after allogeneic hematopoietic stem cell transplantation among recipients with CMV infection

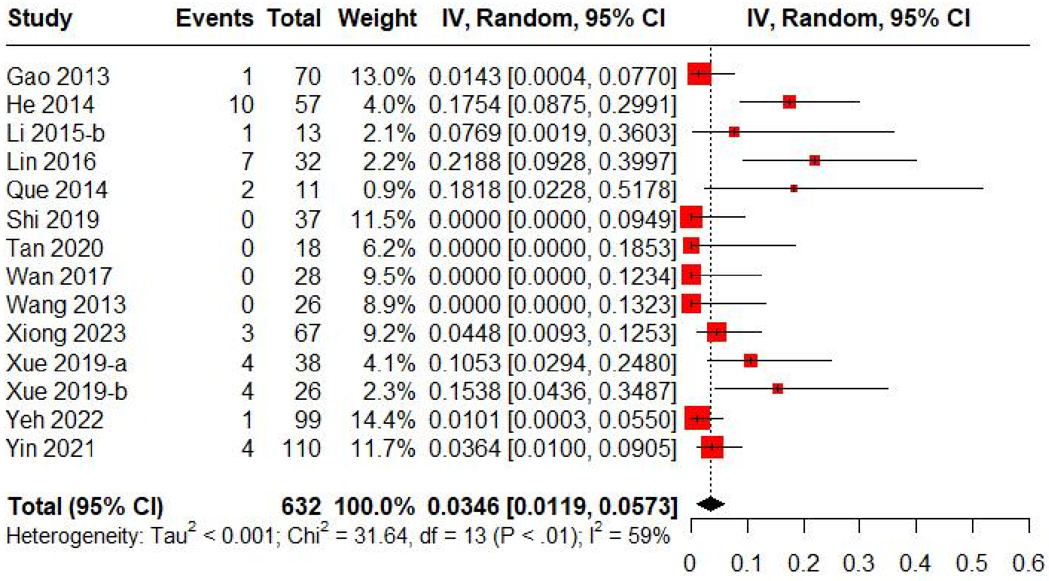

Figure S21 Forest plot of CMV-related mortality after allogeneic hematopoietic stem cell transplantation among recipients with CMV infection

Incidence of comorbidities

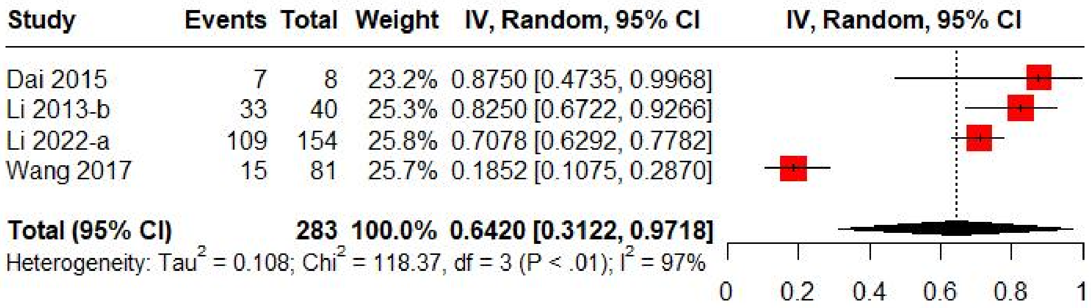

Figure S22 Forest plot of incidence of graft versus host disease after allogeneic hematopoietic stem cell transplantation among recipients with CMV infection

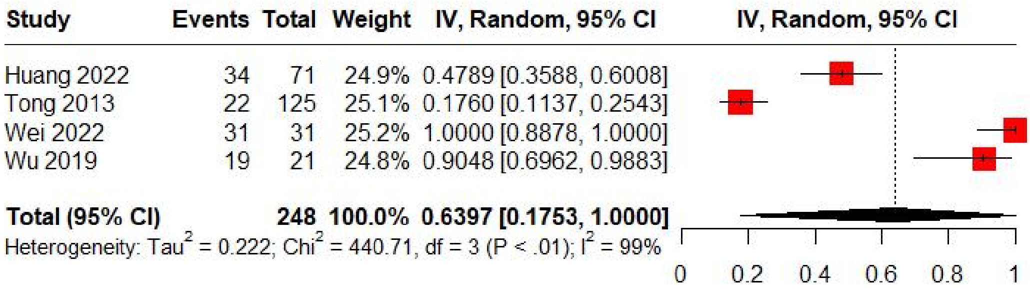

Figure S23 Forest plot of incidence of acute graft versus host disease after allogeneic hematopoietic stem cell transplantation among recipients with CMV infection

Supplementary 4 Funnel plots assessing publication bias on the results with 10 or more included studies

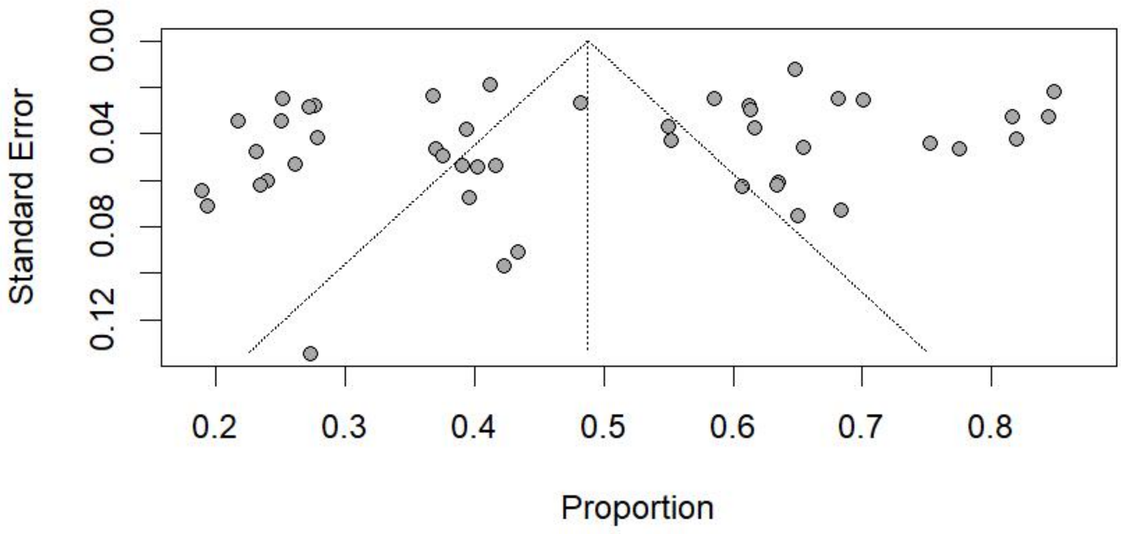

Figure S24 Funnel plot for meta-analysis of CMV infection incidence in recipients after allogeneic hematopoietic stem cell transplantation

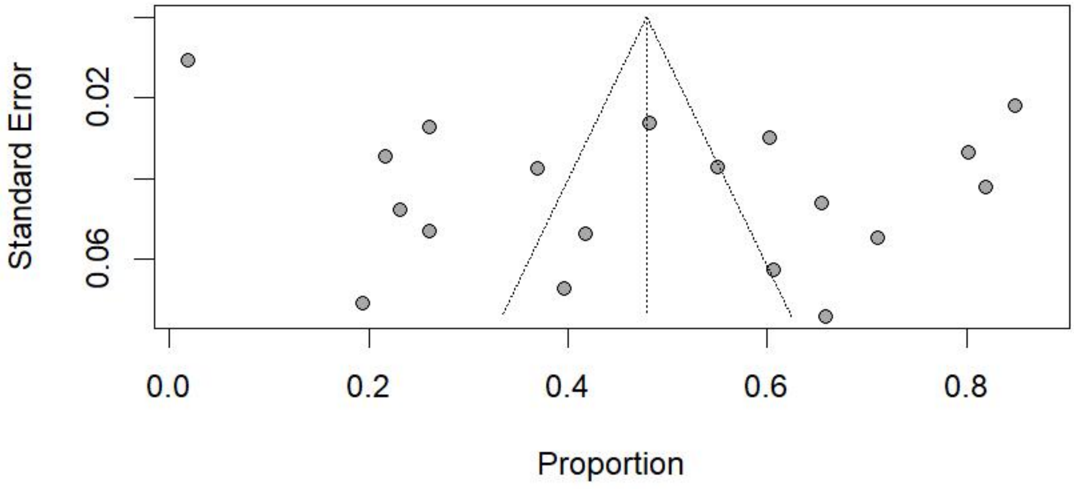

Figure S25 Funnel plot for meta-analysis of CMV infection incidence in recipients within 100 days after allogeneic hematopoietic stem cell transplantation

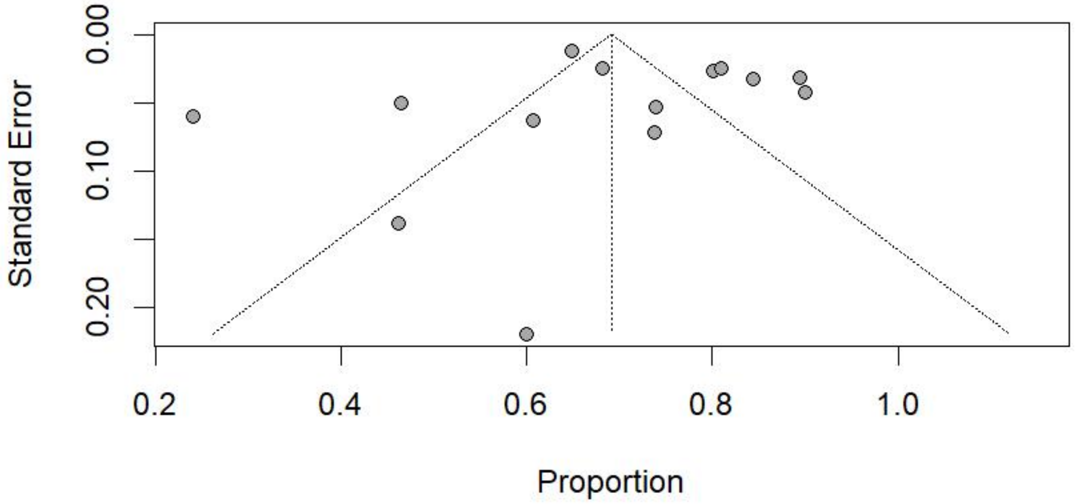

Figure S26 Funnel plot for meta-analysis of CMV infection incidence in recipients after haploidentical allogeneic hematopoietic stem cell transplantation

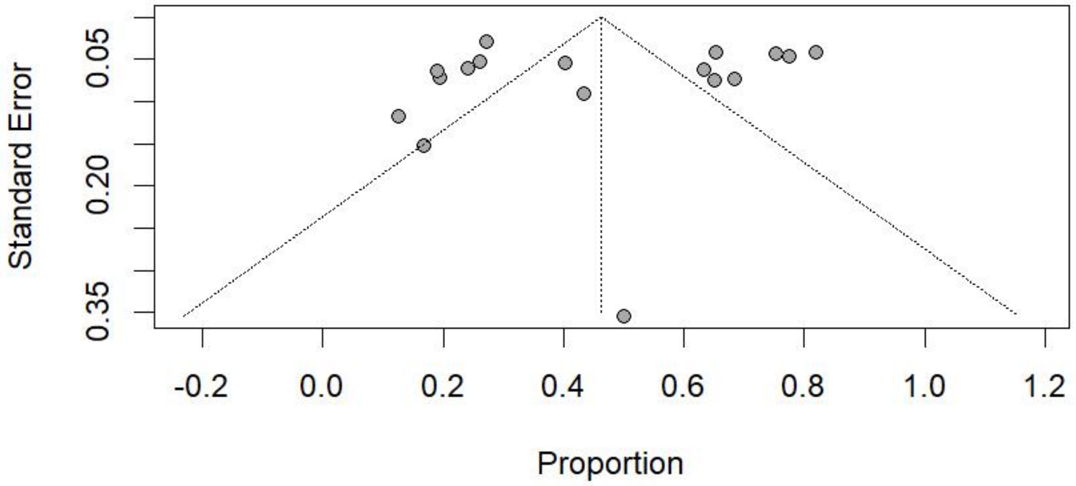

Figure S27 Funnel plot for meta-analysis of CMV infection incidence in recipients after haploidentical allogeneic hematopoietic stem cell transplantation when the CMV serostatus was negative in donor and recipients

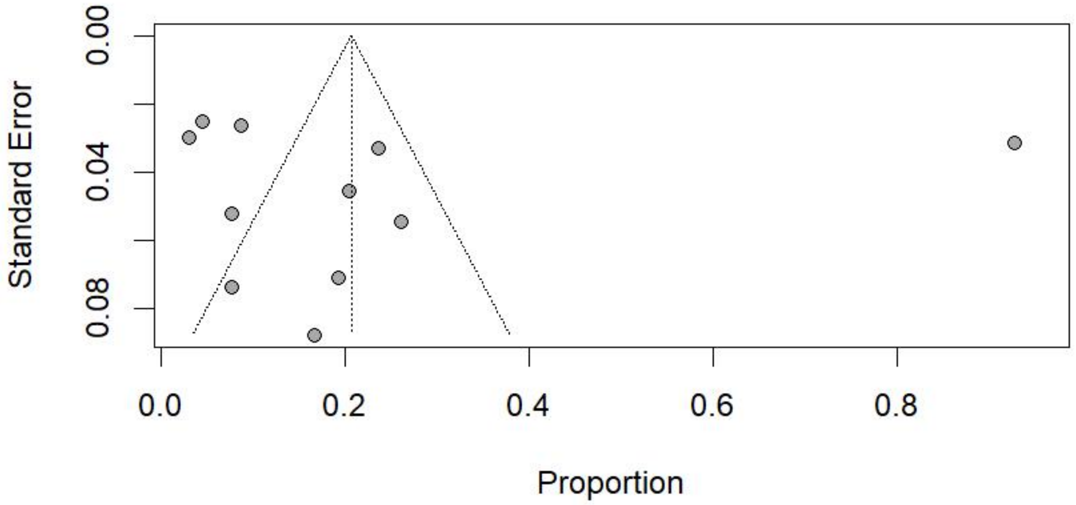

Figure S28 Funnel plot for meta-analysis of recurrent CMV infection incidence in recipients after allogeneic hematopoietic stem cell transplantation

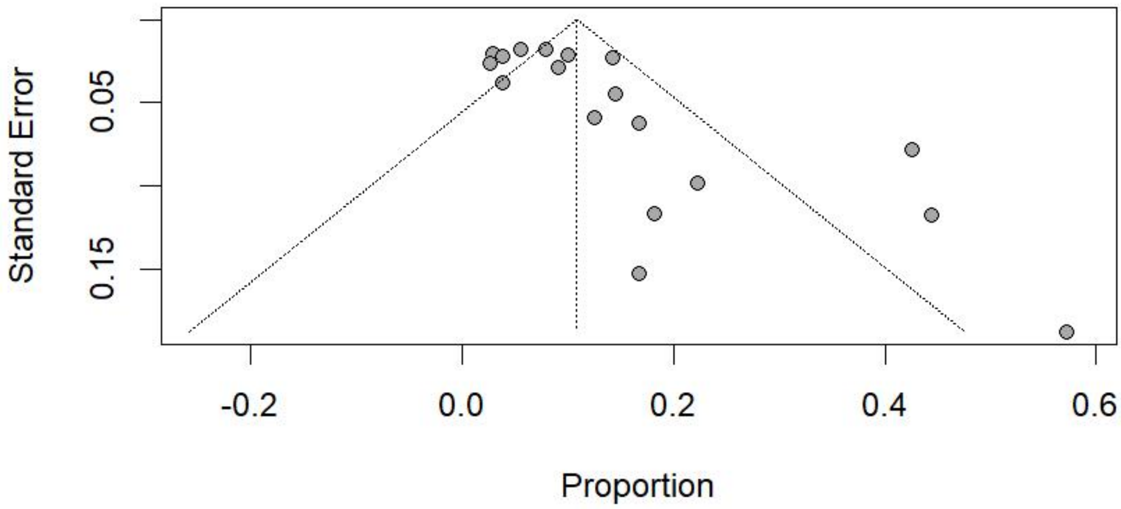

Figure S29 Funnel plot for meta-analysis of CMV disease incidence in recipients with CMV infection after allogeneic hematopoietic stem cell transplantation

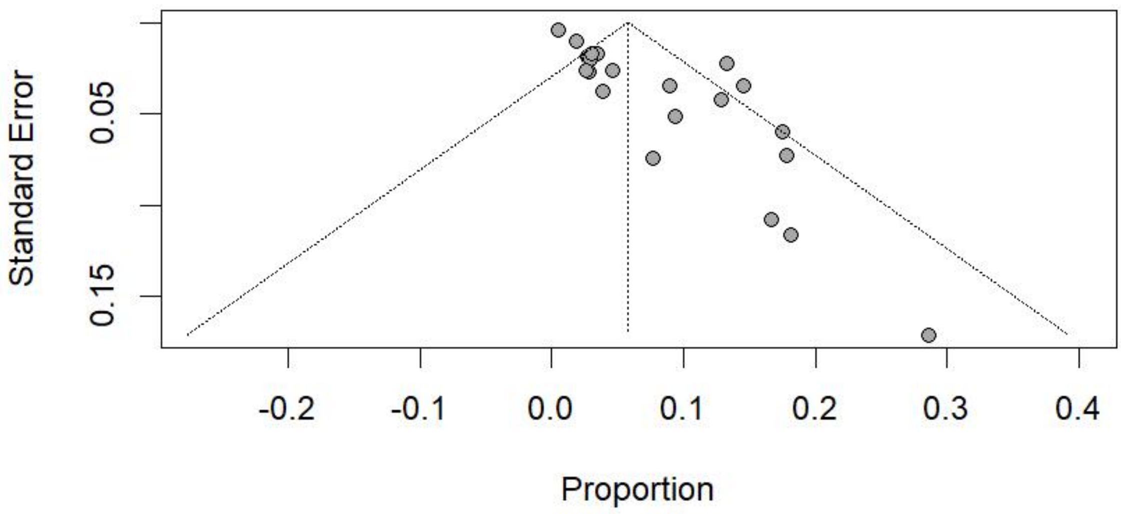

Figure S30 Funnel plot for meta-analysis of CMV pneumonitis incidence in recipients with CMV infection after allogeneic hematopoietic stem cell transplantation

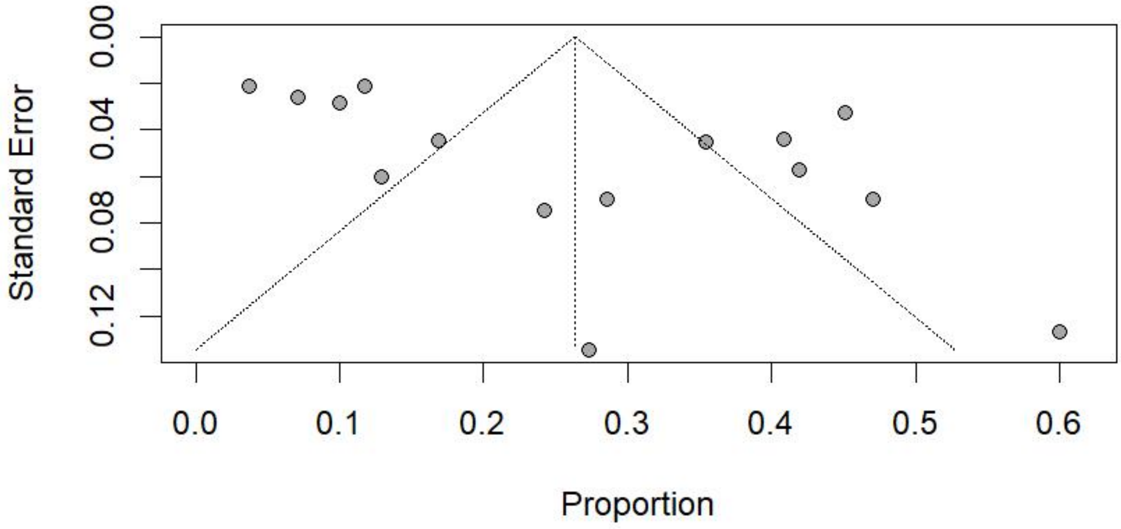

Figure S31 Funnel plot for meta-analysis of all-cause mortality rate in recipients with CMV infection after allogeneic hematopoietic stem cell transplantation

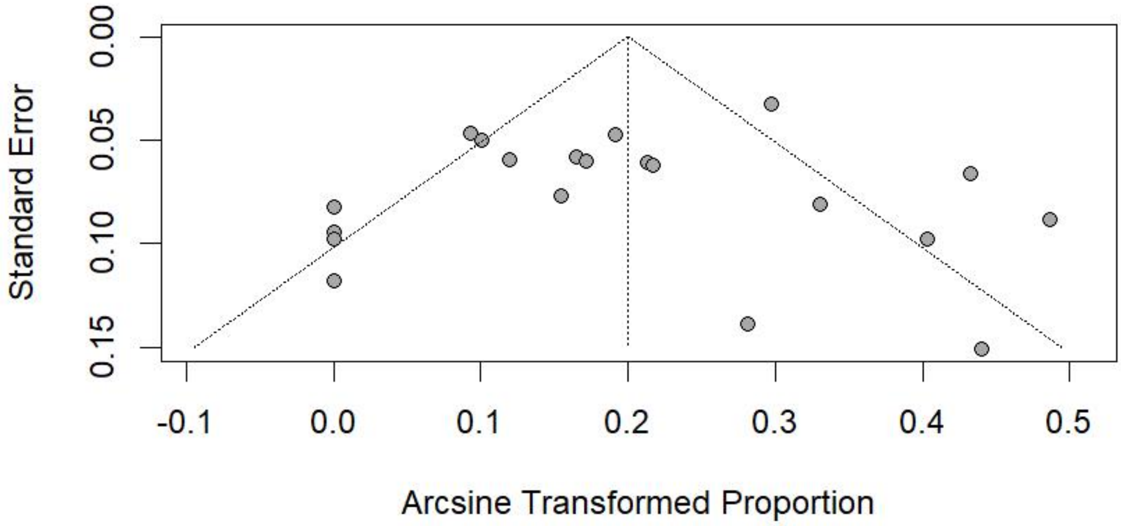

Figure S32 Funnel plot for meta-analysis of CMV-related mortality rate in recipients with CMV infection after allogeneic hematopoietic stem cell transplantation
